# bHLH Transcription Factor Math6 Antagonizes TGF-β Signalling in Reprogramming, Pluripotency and Early Cell Fate Decisions

**DOI:** 10.3390/cells8060529

**Published:** 2019-06-02

**Authors:** Satya Srirama Karthik Divvela, Patrick Nell, Markus Napirei, Holm Zaehres, Jiayu Chen, Wanda Maria Gerding, Huu Phuc Nguyen, Shaorong Gao, Beate Brand-Saberi

**Affiliations:** 1 Ruhr University Bochum, Medical Faculty, Department of Anatomy and Molecular Embryology, 44801 Bochum, Germany; satya.divvela@rub.de (S.S.K.D.); nell@ifado.de (P.N.); Markus.Napirei@rub.de (M.N.); holm.zaehres@rub.de (H.Z.); 2 School of Life Science and Technology, Tongji University, 200092 Shanghai, China; Chenjiayu@tongji.edu.cn (J.C.); gaoshaorong@tongji.edu.cn (S.G.); 3 Leibniz Institut für Arbeitsforschung, Technische Universität Dortmund, 44139 Dortmund, Germany; 4 Ruhr University Bochum, Medical Faculty, Department of Human Genetics, 44801 Bochum, Germany; Wanda.Gerding@rub.de (W.M.G.); huu.nguyen-r7w@rub.de (H.P.N.)

**Keywords:** Atoh8, Math6, reprogramming, MET, primed state, pluripotency, mesendoderm, TGF beta

## Abstract

The basic helix-loop-helix (bHLH) transcription factor Math6 (Atonal homolog 8; Atoh8) plays a crucial role in a number of cellular processes during embryonic development, iron metabolism and tumorigenesis. We report here on its involvement in cellular reprogramming from fibroblasts to induced pluripotent stem cells, in the maintenance of pluripotency and in early fate decisions during murine development. Loss of Math6 disrupts mesenchymal-to-epithelial transition during reprogramming and primes pluripotent stem cells towards the mesendodermal fate. Math6 can thus be considered a regulator of reprogramming and pluripotent stem cell fate. Additionally, our results demonstrate the involvement of Math6 in SMAD-dependent TGF beta signalling. We furthermore monitor the presence of the Math6 protein during these developmental processes using a newly generated *Math6Flag-tag* mouse. Taken together, our results suggest that Math6 counteracts TGF beta signalling and, by this, affects the initiating step of cellular reprogramming, as well as the maintenance of pluripotency and early differentiation.

## 1. Introduction

Atonal homolog 8 (Atoh8), that is, Mouse atonal homolog 6 (Math6), belongs to the group of basic helix loop helix (bHLH) transcription factors, which regulate the expression of genes required for cell commitment and differentiation. The bHLH transcription factors in general function either as transcriptional activators or repressors [1,2] and play important regulatory roles during developmental processes, such as myogenesis, neurogenesis and haematopoiesis, as well as the development of the gastrointestinal and reproductive tract [3]. Atoh8 possesses two highly conserved and functionally distinct basic DNA-binding domains, which are followed by two alpha–helices separated by a variable loop region (HLH). The HLH region thereby facilitates the formation of homo- and heterodimers [4].

*Math6* was first described in the context of neurogenesis where it was found to be expressed in neuronal precursor cells of the ventricular zone and subsequently in differentiating neurons [5]. Later, like other bHLH transcription factors, Math6 was identified in the commitment and differentiation of multiple developmental processes including that of the pancreas [6], kidney [7], skeletal muscles [8], heart [9] and placenta [10]. Recently, our group performed a systematic study to identify the expression profile of Math6 in early and late developmental stages of the mouse. The expression of Math6 in late developmental stages correlates with the current literature described above [11]. Nevertheless, our spatiotemporal investigations also implied a function for Math6 during early embryogenesis. Thus, we were able to detect its expression within the inner cell mass of blastocysts, which is built up of pluripotent embryonic stem cells (ESCs). In addition, we could reveal a translocation of Math6 from the cytoplasm into the nucleus of ESCs, an observation which underlines its function as a transcription factor with significance for either the maintenance of their stem cell property or even their differentiation [11].

Contradictory literature exists with regard to Atoh8, as it was first described as a possible oncogene based on its copy number in a study performed on glioblastoma stem cells [12]. Subsequently, *Atoh8* was found to be differentially regulated in renal carcinoma cells [13] and glioblastoma stem cells treated with retinoic acid [14]. A study performed on hepatocellular carcinomas (HCC), however, emphasized Atoh8 as a potential tumour suppressor gene, the absence of which imparts stem cell properties to cancer cells. Ectopic expression of *Atoh8* in HCC cell lines disrupts their proliferation, foci colony formation, invasive and migratory abilities. Furthermore, a reprogramming assay performed with human fibroblasts revealed an enhanced reprogramming, which was accompanied by the depletion of *Atoh8* expression. Accordingly, Atoh8 was shown to downregulate the transcription of the pluripotency factors Oct4 and Nanog [15]. In 2016, another study performed on nasopharyngeal carcinomas showed that the inhibition of *Atoh8* enhanced the mesenchymal status and contributed to the malignant phenotype [16]. In the same study, the inhibition of *Atoh8* led to the downregulation of the epithelial marker E-cadherin and upregulation of the mesenchymal marker vimentin. Atoh8 has thereby been reported as a potential regulator of epithelial-to-mesenchymal transition (EMT), which has been proposed as a major initiator of metastasis [16]. Lately, study performed on human ESCs revealed *Atoh8* as a shear stress-responsive gene [17]. Atoh8 was described as a pivotal transcription factor in the determination of endothelial precursor cells. These authors also reported that neither loss nor gain of function studies regarding *Atoh8* altered the expression of the core pluripotent markers *Oct4, Sox2* and *Nanog* [17], a finding which is in clear contrast to the data published by Song’s group.

Considering that Math6 is widely expressed during murine embryonic development [11] and that recent studies on human cancer describe Atoh8 as a tumour suppressor gene with a potential influence on EMT, its spatiotemporal expression along with genes maintaining the pluripotent property of murine ESCs raises several questions concerning its role in determining pluripotency or early differentiation. To characterize the role of Math6 further, we therefore generated a constitutive *Math6* knockout mouse and performed loss of function studies [10]. Due to the lack of specific anti-Math6 antibodies, a *Math6Flag-tag* reporter mouse was generated in addition to substantiate the spatiotemporal expression pattern of *Math6*.

In the current study, we have evaluated the role of Math6 in somatic cell reprogramming, maintenance of pluripotency and early stem cell differentiation.

## 2. Materials and Methods

### 2.1. Successful Generation of Math6Flag-Tag Mice using CRISPR-CAS9 Technology

In the present study, we used human codon-optimized Cas9 from Streptococcus pyogenes in combination with an optimized sgRNA scaffold to target exon 3 of the murine Math6 gene by knock-in of a 3xFLAG sequence. The recombinant expression of C-terminally flagged Math6 should solve the problem of a lack of specific anti-Math6 antibodies and enable a highly reliable spatiotemporal analysis of Math6 by anti-Flag immunodetection (Figure S1). Candidate crRNA sequences were obtained from crispr.mit.edu and cloned into the sgRNA scaffold of the plasmid pSLQ1651-sgTelomere (F + E) provided by Bo Huang and Stanley Qi labs (Figure S2). Cas9 mRNA and sgRNA for Math6 targeting were produced by in vitro transcription (IVT) and injected into BDF1 zygotes obtained from female C57BL/6 mated to male DBA2 mice (Figures S3 and S4). Successful targeting of Math6 by candidate crRNA sequences was evaluated by employing the T7 endonuclease assay, which detects mismatches between DNA isolated from manipulated blastocytes and that of C57BL/6 wild-type mice (Figure S5). A knock-in donor construct was designed for integration at the sgRNA target site, producing an in-frame knock-in of the 3xFLAG sequence directly upstream of the Math6 stop codon in exon 3 (Figure S6). Donor construct integration at the Math6 gene locus of transgenic mice was validated by PCR genotyping and DNA sequencing (Figure S7).

### 2.2. Cell Culture

Mouse adult fibroblasts (MAFs) and embryonic fibroblasts (MEFs) were isolated as described [18,19] respectively. Embryonic fibroblasts, adult fibroblasts and HEK cells were maintained in DMEM, 10% FBS, 1% L-Glutamine, 1% NEAA and 1% P/S. Reprogramming was performed using the following media supplemented with DMEM, 15% FBS, 1% L-Glutamine, 1% NEAA, 1% P/S, 0.1mM 2-mercaptoethanol and 1000U/mL of Leukemia inhibitory factor (LIF) (STEMCELL Technologies, Grenoble, France). Induced pluripotent stem cells (iPSCs) and Embryonic stem cells (ESCs) were maintained in 2i medium [20]. For differentiation of iPSCs and ESCs, DMEM/F12, 15% KOSR, 1% L-Glutamine, 1% NEAA, 1% P/S and 0.1mM 2-mercaptoethanol was used. TGFb1 (Peprotech, Hamburg, Germany) was used at 5ng/mL concentration, SB-431542 (STEMCELL Technologies, Grenoble, France) was used at a final concentration of 1µM.

### 2.3. Generation of Mouse iPS Cells

Retrovirus vectors encoding Oct4, Sox2, Klf4, cMyc (Addgene plasmids 13366, 13367, 13370, 13375) and Green fluorescent protein (GFP) were produced as ecotropic viruses with pCL-Eco (Addgene plasmid 12371) in HEK293 cells. Following co-transfection, viral supernatants were collected after 48 and 72 h and were subjected to ultracentrifugation at 25,000 rpm. 100.000 fibroblasts (passage 2) were transduced in media supplemented with polybrene at 8µg/mL. A timeline of the reprogramming experiments is provided in Supplementary information 2.

### 2.4. Alkaline Phosphatase Staining

Alkaline phosphatase (AKP) staining was performed using the manufacturer’s instructions-Merck Millipore, Darmstadt, Germany (SCR004). AKP Staining was performed on Day 14 for the cells reprogramming on feeder cells and on day 20 for the cells reprogramming on Matrigel (Corning, Amsterdam, The Netherlands).

### 2.5. Calculation of Reprogramming Efficiency

Reprogramming efficiency was calculated as the ratio of a total number of colonies observed on day 14 or day 20 to the total number of fibroblasts infected and is displayed as a percentage.

### 2.6. Isolation of Mouse Blastocysts

Blastocysts from wildtype (WT) and knockout (KO) C57Bl/6J mice were isolated and ESCs were established following the protocol described by Behringer [21].

### 2.7. Culturing iPSCs and ESCs

iPSCs and ESCs were cultured in 2i/LIF media. Cells were passaged once every four days using TRYPLE reagent (Invitrogen). The seeding density used during the passage is 1000 cells/cm^2^. Rock inhibitor was supplemented to the media for the first 12 h after passage.

### 2.8. Chromosomal Analysis

Karyotype analysis of (3 karyogrammes were analysed/counted per culture) G-banded metaphase spreads from iPS and ES cell culture samples were performed using standard methods. Karyotypes were analysed with the Isis and Ikaros Karyotyping software (Metasystems, Altusheim, Germany) and revealed normal 40 XY mouse karyotypes.

### 2.9. Morphometric Analysis

iPS cell lines of both WT and KO were plated at a seeding density of 2000 cells/cm^2^ on Matrigel-coated Thermanox© coverslips in ‘2i’ media. After 24 h, the cells were fixed in 2.5% glutaraldehyde overnight at 4 °C. Following fixation, cells were dehydrated using acetone and were embedded in Durcupan. The embedded cells were then subjected to sectioning using microtome generating semi-thin and ultra-thin sections. Semi-thin sections were stained with methylene blue. Ultra-thin sections were used to generate Transmission electron microscopy pictures. Semi-thin sections were used to study and analyse cell-cell contacts.

### 2.10. RNA isolation, Reverse Transcription and Real-time PCR (RT-PCR)

RNA was isolated using TRI reagent (Sigma-Aldrich, Munich, Germany), cDNA synthesis using GoScript Reverse transcriptase—Promega; Real-time quantification was performed using GoTaq qPCR master mix—Promega, Mannheim, Germany. These reactions were performed following the respective manufacturer’s instructions. The Livak method was used to calculate relative quantification.

### 2.11. Immunostaining

Cells grown on 4-well plates were washed thrice with PBS, followed by fixation with 4% paraformaldehyde for 15 min. The cells were then permeabilized with 0.5% Triton X-100 for 15 min and blocked using 5% BSA for 30 min at room temperature. Following blocking, cells were incubated with primary antibody at 4 °C overnight. Next morning cells were washed thrice with 1xPBS and incubated with secondary antibody for 1 h. Following this, the cells were washed thrice with 1xPBS and mounted using mounting media containing DAPI.

### 2.12. Western Blot

The Trizol protein isolation method was used to extract protein from cells grown on a 35 mm dish. Isolated protein was quantified using Bradford’s assay. For all protein detections 50 µg of protein was used but for Math6 - Flag detection 100 µg of protein was loaded on 12.5% SDS-Page gel. Western blot was performed according to Abcam Western blot protocol. Following blotting, the blots were incubated with appropriate primary antibodies at 4 °C overnight. Later, the blots were incubated with appropriate HRP conjugated secondary antibodies at room temperature for an hour. ECL reagent was used for imaging the blots.

### 2.13. Statistical Analysis

The data presented are mean ± SD. Sample numbers and repeats are given in figure legends. Statistical significance was determined by the Holm Sidak Method t-test using GraphPad Prism 8, La Jolla, California, USA Differences in means were shown statistically significant at *p* < 0.05. Significance levels are shown as *(ns) no significance p >* 0.05, * *p* ≤ 0.05, ** *p* ≤ 0.01 and *** *p* ≤ 0.001.

## 3. Results

### 3.1. Math6 Expression during Somatic Cell Reprogramming

To evaluate the expression of Math6 during somatic cell reprogramming, we prepared fibroblasts from the ears of adult *Math6Flag-tag* mice and subjected them to reprogramming using Oct4, Sox2, Klf4 and c-Myc (OSKM) [22]. Mechanistically, based on transcriptomic profiling, reprogramming has been divided into an early initiation, an intermediate maturation and a late stabilization phase [23]. In the current study, we have chosen one timepoint from each of the different phases to evaluate the expression of Math6 and the genes that are involved in reprogramming (Figure S8).

**Figure 1:**
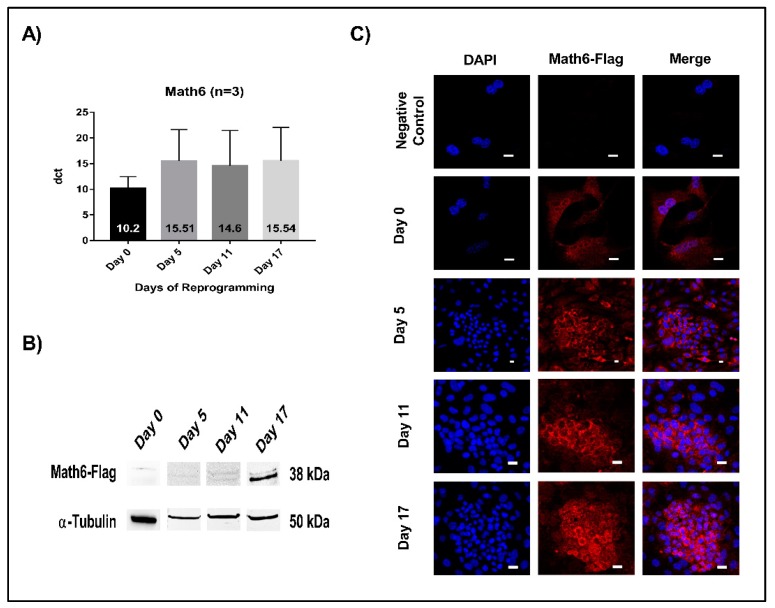
Math6 expression during somatic cell reprogramming. (**A**) mRNA expression of *Math6* on Day 0 (Untransduced fibroblasts), 5, 11 and 17 of reprogramming generated by RT-PCR. The data shown were normalized to housekeeping gene *GAPDH*. The data shown is the average of three similar individual sets of experiments. From here on this applies to all gene expression data. (**B**) Expression of Flag-tagged Math6 on Day 0, 5, 11 and 17 of reprogramming analysed by Western blot. α-tubulin was used as a control. (**C**) Immunofluorescence images showing Flag-tagged Math6 expression in fibroblasts, Day 0, 5, 11, 17 of reprogramming.

Expression of *Math6* was analysed both at the mRNA and protein level using quantitative RT-PCR and Western blot, respectively. The timepoints signify as follows: day 5 (initiation), day 11 (maturation) and day 17 (stabilization phase). The mRNA levels of *Math6* were found to be equal during the different phases of reprogramming (Figure 1A). However, at the protein level, Math6 was found increasingly detectable as the cells progress towards the induced pluripotent stem cell (iPSC) state (Figure 1B). Concordantly, anti-Flag immunostaining revealed an upsurge in the presence of Math6-Flag particularly in the committed reprogramming cells (Figure 1C).

### 3.2. Math6 Promotes Somatic Cell Reprogramming

**Figure 2:**
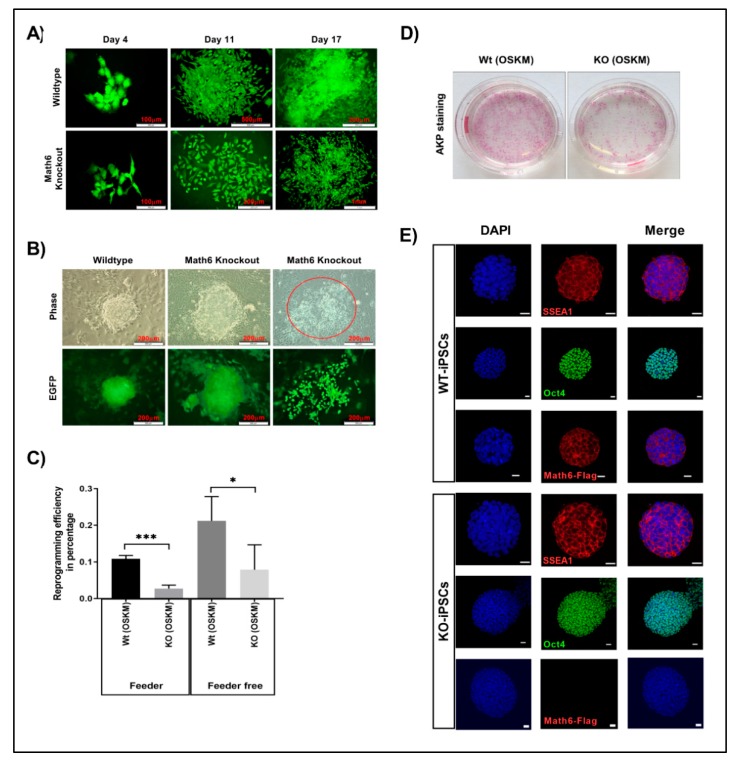
Math6 promotes somatic cell reprogramming. (**A**) Comparison of wildtype and Math6 knockout reprogramming on the feeder. Wildtype (WT) cells could undergo reprogramming on the feeder, whereas knockout (KO) cells failed to undergo proper reprogramming on the feeder. (**B**) Comparison of wildtype and Math6 knockout reprogramming performed on Matrigel on Day 14. Both WT and KO fibroblasts could undergo reprogramming. However, in Math6 knockouts we can see both successfully reprogramming colonies and cells that failed to form colonies with dispersed morphology (marked red). (**C**) Reprogramming efficiencies of reprogramming performed on feeder and Matrigel are shown. The statistical significance was determined by the Holm Sidak method t-test using Prism 8. Significance levels are shown as * *p* ≤ 0.05, ** *p* ≤ 0.01 and *** *p* ≤ 0.001. Hereafter, the statistical significance shown in this manuscript follows the same. (**D**) Alkaline phosphatase staining showing reprogramming efficiency in wildtype and Math6 knockout on Day 20 of reprogramming (Feeder-free). (**E**) Immunofluorescence images of iPS cell lines derived by reprogramming fibroblasts as follows WT (OSKM), KO (OSKM). iPSC colonies positive for SSEA1 (red), Oct4 (green) and Math6—Flag (red) and DAPI (blue) are shown. Scale bar indicates 20µm.

In order to ascertain the role of Math6 in somatic cell reprogramming, fibroblasts derived from wildtype (WT) and Math6 knockout (KO) mice were transduced with OSKM along with green fluorescent protein (GFP). Reporter GFP was used to distinguish reprogramming adult fibroblasts from feeder cells. Transduced adult fibroblasts were transferred onto mitomycin-C-treated feeder cells and culture medium was supplemented with leukaemia inhibitory factor (LIF). Wildtype fibroblasts were able to undergo proper reprogramming with a reprogramming efficiency (RE) of 0.1% (Figure 2A,C). However, KO fibroblasts appeared to have diminished acquisition of epithelial characteristics and in turn led to the failure of reprogramming (RE = 0.02%) (Figure 2A,C). In the next step, to understand the reason for the failure of Math6 KO fibroblasts to undergo reprogramming and to evaluate the influencing factors derived by the cell culture conditions, we reprogrammed fibroblasts under feeder-free conditions on Matrigel©. Again, only a minority of KO fibroblasts were able to obtain epithelial characteristics and could further attain pluripotent cell morphology (Figure 2B–D). The cells that failed in reprogramming showed a dispersed morphology implying the lack of proper cell-cell contacts (Figure 2B). However, the reprogramming efficiency of WT and KO fibroblasts under feeder-free conditions improved by 2-fold (0.2%) and 4-fold (0.08%), respectively (Figure 2C). These data imply that indeed two important antagonistic factors influence the efficiency of reprogramming: on the one hand Math6 as an activator and on the other side a so far unidentified repressor derived by the culture conditions. The iPSCs generated from WT and KO reprogramming are shown in (Figure 2E).

### 3.3. Lack of Math6 Disrupts Mesenchymal-to-Epithelial Transition (MET) and Somatic Cell Reprogramming

**Figure 3:**
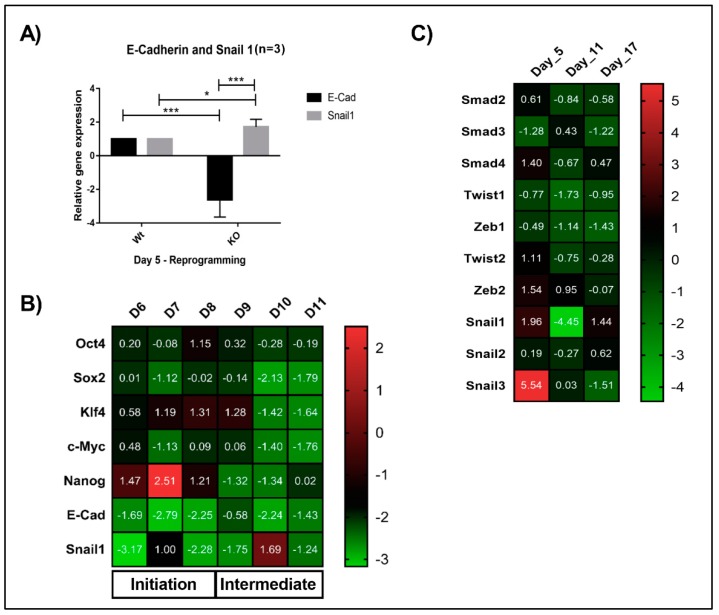
Lack of Math6 disrupts mesenchymal-to-epithelial transition (MET) and somatic cell reprogramming. (**A**) Relative gene expression of the epithelial marker *E-cadherin* and mesenchymal marker *Snail1* on day 5 of WT and KO reprogramming cells. The data presented is normalized to *GAPDH*. This is applicable to all the gene expression data shown in this figure. (**B**) Expression of Yamanaka’s factors (*OSKM*), *Nanog*, MET markers (*Snail1 and E-cadherin*) during the reprogramming from day 6-11 (*n* = 3). (**C**) Expression of pro-mesenchymal genes during different phases of KO reprogramming cells on Day 5, 11 and 17 (*n* = 3). Statistical significance levels were shown as * *p* ≤ 0.05 and *** *p* ≤ 0.001.

The Math6 KO fibroblasts cultured in the absence of feeder cells showed an enhanced reprogramming efficiency (4-fold) compared to the cells cultured on the feeder layer. Mouse embryonic feeder cells in addition to LIF also tend to secrete high levels of TGF-β and Activin-A which are master regulators of epithelial-to-mesenchymal transition (EMT) that is, pro-mesenchymal factors [19]. Based on the morphological differences observed for KO fibroblasts under feeder and feeder-free culture conditions (Figure 2A,B), we speculated that the failure of KO fibroblasts to undergo MET and reprogramming on a feeder layer might be, in addition to the lack of Math6, caused by pro-mesenchymal signals provided by the culture conditions (feeder cells, foetal bovine serum). Following OSKM transduction, reprogramming cells gain pronounced cell-cell contacts via the activation of epithelial genes while inhibiting pro-mesenchymal genes. Subsequently, in the next stage, they activate developmental and pluripotency-associated genes before achieving the pluripotent state [24]. To gain more insight into the function of Math6, the adult fibroblasts cultured under feeder-free conditions were subjected to transcriptional profiling during reprogramming. Mesenchymal epithelial transition markers E-cadherin and Snail1 were studied on Day 5, which revealed the downregulation of *E-cadherin* and upregulation of *Snail1* (Figure 3A). Following this, we have systematically checked the expression of pluripotency-associated genes (*Oct4, Sox2, Klf4, c-Myc* and *Nanog*), MET markers (*E-cadherin and Snail1*) from Day 6 to Day 11 of reprogramming, the data obtained revealed a consistent downregulation of epithelial marker *E-cadherin* and differential expression of *Snail1*. At the same time, we have also observed the downregulation of pluripotency associated markers from Day 10 of reprogramming suggesting that the lower reprogramming efficiency in Math6 knockouts is indeed due to the failure in the acquisition of epithelial characteristics (Figure 3B). Furthermore, to confirm this, tests were performed to check the status of pro-mesenchymal genes (*Smad 2, 3,* & *4, Twist 1* & *2, Zeb 1* & *2, Snail 1, 2* & *3*) at three different time points that corresponded to the different phases of reprogramming. Gene expression data of Math6 KO cells revealed an upregulation of *Smad4, Zeb2 and Snail 1* & *3* (Figure 3C). These findings prompted us to regard Math6 as a promoting regulator of MET which is required for the initial step of somatic cell reprogramming.

### 3.4. Math6 Enables MET by Antagonizing TGF-β Signalling

**Figure 4:**
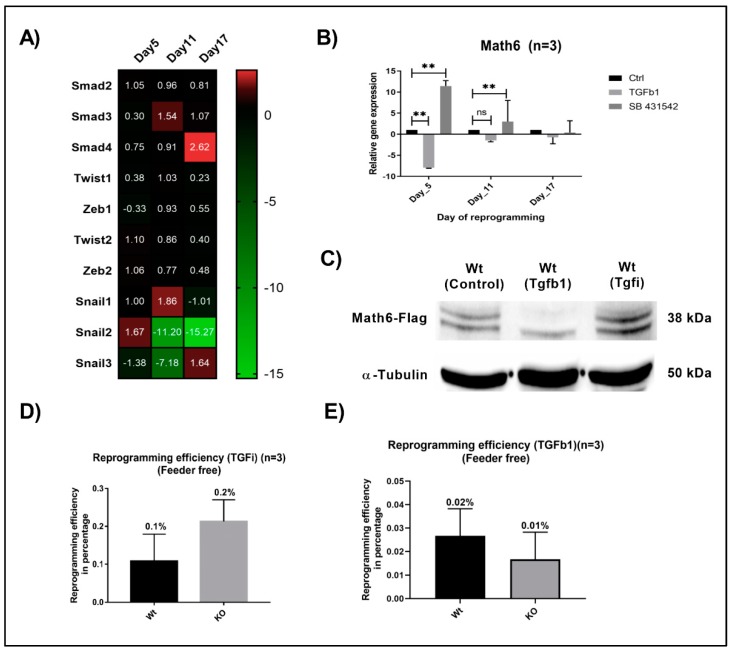
TGF-β signalling counteracts the promoting effect of Math6 on MET and somatic cell reprogramming. (**A**) Expression of pro-mesenchymal genes during different phases of KO reprogramming cells treated with SB-431542 (*n* = 3). (**B**) Expression of *Math6* during different phases of reprogramming in control reprogramming, TGFb1 treated reprogramming and SB-431542 treated reprogramming. We observed a significant downregulation of *Math6* after TGFb1 treatment on day 5. Similarly, inhibition of TGF-β significantly upregulated *Math6* expression on day 5 and 11. Statistical significance levels were shown as *(ns*) no significance *p* > 0.05, and ** *p* ≤ 0.01, (C) Western blot analysis showing the presence of Math6 *Flag* on day 5 of reprogramming. It shows the response of Math6 protein levels with changes in the TGF-β signalling. (**D**) The efficiency of reprogramming calculated in WT and KO with SB-431542 treatment. The reprogramming process has been improved in KO (0.2%) compared to WT (0.1%). (**E**) The efficiency of reprogramming calculated in WT and KO with TGFb1 treatment. The reprogramming process was severely damaged and resulted in poor reprogramming efficiency in both WT (0.02%) and KO (0.01%) cells.

Previous studies have emphasized TGF-β signalling as a potent activator of EMT and repressor of MET through TGF-β type I receptors called activin receptor-like kinases (ALK4, 5 and 7) [25]. To investigate the connection of Math6 with MET and TGF-β signalling in reprogramming, we treated the reprogramming fibroblasts with a selective inhibitor of TGF-β signalling called SB-431542, which has been proven to act by inhibiting the phosphorylation of the receptors Alk4, 5 and 7 [26]. The fibroblasts cultured in the absence of a feeder layer were treated with SB-431542 from day 4 to day 6 of reprogramming. Afterwards, the reprogramming cells were subjected to transcriptional profiling for pro-mesenchymal markers. Compared to the WT, we unexpectedly observed downregulation of pro-mesenchymal *Smad4, Zeb2, Snail1* and *Snail3* in reprogramming Math6 KO cells on day 5 under TGF-β inhibition although the repressing effect of TGF-β signalling on MET should be reduced equally in both, WT as well as KO fibroblasts (Figure 4A). Interestingly, we observed an upregulated *Math6* mRNA expression of 11.43 and 3.01 folds on day 5 and day 11 in WT reprogramming cells under TGF-β inhibition. This finding could be verified on the protein level at day 5 of reprogramming (Figure 4B,C). However, Math6 upregulation did not result in an improved reprogramming efficiency of WT fibroblasts under TGF-β inhibition in comparison to those cultured in the presence or absence of feeder cells that is, decreasing TGF-β concentrations (Figure 2C and Figure 4D).

Instead, we clearly detected an intriguing decrease of the reprogramming efficiency when comparing fibroblasts under ectopic TGFb1 treatment during day 4 to day 6 of reprogramming with those under inhibition of TGF-β signalling. We found that TGFb1 treatment severely affected the reprogramming process leading to an efficiency of 0.02% for WT and 0.01% for KO fibroblasts (Figure 4E). This finding correlates well with studies on TGF-β signalling as a negative regulator of MET and reprogramming [27]. At the same time, treatment with TGFb1 resulted in a 7.95 and 1.5 folds downregulation in the expression of *Math6* on day 5 and day 11 in reprogramming WT fibroblasts (Figure 4B,C). Indeed, when comparing the reprogramming efficiency of WT and KO fibroblasts under ectopic TGFb1 treatment no significant difference could be observed anymore. Taken together, these data imply that the MET promoting effect of Math6 is antagonized by TGF-β signalling at different degrees depending on the culture conditions. (Figure 2C and 4D).

### 3.5. Math6 Sustains the Naive Pluripotent State of iPSCs

**Figure 5:**
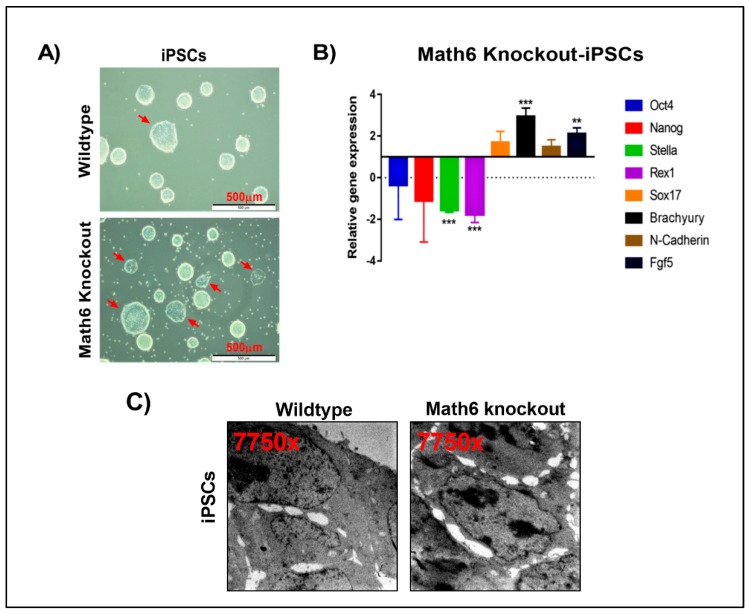
Math6 sustains the naïve pluripotent state. (**A**) WT and KO iPSCs maintained in 2i/LIF medium. KO-iPSCs show more primed colonies. (**B**) Expression of pluripotent markers (*Oct4* & *Nanog*), naive markers (*Stella* & *Rex1*) and primed state markers (*Sox17, Brachyury, N-Cadherin, Fgf5*) in KO-iPSCs. The gene expression shown is normalized to *18s* and relative to WT-iPSCs. Statistical significance levels are shown as ** *p* ≤ 0.01 and *** *p* ≤ 0.001. (**C**) Transmission electron microscope images showing WT and KO iPSCs. KO-iPSCs show poor cell-cell contacts compared to WT-iPSCs.

To investigate the significance of Math6 in the maintenance of pluripotency, we gradually changed the culture conditions of the iPSCs from serum to serum-free by adopting 2i/LIF medium. It has been proven that 2i/LIF medium efficiently maintains the naive state of ES-cells [20]. Naive pluripotent colonies are easy to recognize in cell culture by their bright borders and compact dome-shaped morphology. Compared to WT cultures, we observed a smaller number of naive colonies in Math6 KO iPSC cultures. Many of the colonies appeared flat with no bright borders (Figure 5A). The flat morphology is a characteristic feature of the primed pluripotent state. To investigate further, we subjected both, WT and KO derived iPSCs maintained in 2i/LIF medium, to transcriptional profiling. The transcriptional profiling data revealed an upregulated expression of the prime state markers *Sox17, Brachyury, N-Cadherin, Fgf5* with a simultaneous downregulation of the naive pluripotent markers *Stella* and *Rex1* in Math6 KO iPSCs (Figure 5B). Furthermore, morphometric analysis performed on WT and KO iPSCs also revealed a poor cell-cell contact in case of KO-iPSCs (Figure 5C & S12). This clearly demonstrates the significance of Math6 in safeguarding the naive pluripotent state.

### 3.6. Isolation and Stabilization of WT and Math6 KO ESCs

After the isolation of blastocysts from WT and Math6 KO mice, we plated them in cell culture dishes coated with mitomycin-C treated mouse embryonic feeder (MEF) cells. Both WT and KO blastocysts hatched between 48–72 h exposing their inner cell mass (ICM). From this timepoint onwards, the medium was changed partially every 24 h. The WT-derived ICM proliferated normally and formed a colony around day 7, whereas in Math6 KO blastocysts, the ICM seemed to differentiate readily without proliferation and formation of a colony. WT-ESC colonies were picked and expanded according to standard procedures (Figure S9). Considering the phenotype of KO iPSCs, we then tested the effect of avoiding MEF cells and foetal bovine serum. Therefore, we plated Math6 KO blastocysts onto Matrigel© coated dishes filled with knockout serum replacement (KOSR) based medium. The blastocysts hatched normally between 48–72 h but the ICM still failed to proliferate and to form a colony. At last, we tested supplementing 2i/LIF-medium at lower concentrations (25% increment every day) along with KOSR based medium. In this case, we finally observed a proliferating ICM and obtained a Math6 KO-ESC colony around day 7. We picked the observed colony and expanded the clone in 2i/LIF medium (Figure S9). Once again, these observations strongly support the significance of Math6 in the maintenance of pluripotency.

### 3.7. Lack of Math6 Primes Pluripotent Stem Cells into the Mesodermal Lineage

**Figure 6:**
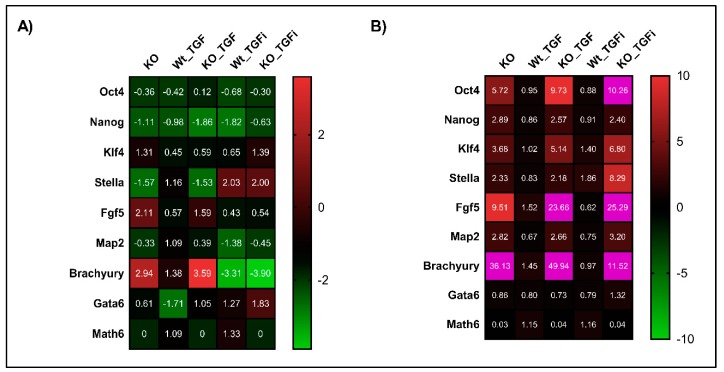
Math6 counteracts TGF-β signalling to restore naive pluripotent state. (**A**) Expression of pluripotency markers (*Oct4* & *Nanog*), primed state markers (*Klf4, Stella* and *Fgf5*) and differentiation markers (*Map2, Brachyury* and *Gata6*) and Atoh8 in KO-iPSCs, WT-iPSCs treated with TGFb1, KO-iPSCs treated with TGFb1, WT-iPSCs treated with SB-431542 and KO-iPSCs treated with SB-431542. The gene expression shown is normalized to *18s* and relative to WT-iPSCs. (**B**) Expression of pluripotency markers, primed state markers and differentiation markers in KO-ESCs, WT-ESCs treated with TGFb1, KO-ESCs treated with TGFb1, WT-ESCs treated with SB-431542 and KO-ESCs treated with SB-431542. The gene expression shown is relative to *18s* and normalized to WT-ESCs. The gene expression data showing more than 10-fold are depicted in pink in the heat map.

To understand the impact of Math6 for maintaining pluripotency of stem cells, WT- and KO- derived iPSCs and ESCs were cultured in 2i/LIF medium and the gene expression profile of pluripotency and differentiation markers were analysed. The data received revealed an upregulation of the mesodermal marker *Brachyury* in both KO-iPSCs and ESCs suggesting their priming and commitment to the mesodermal lineage in the absence of Math6 (Figure 6A,B). In contrast to KO-iPSCs, KO-ESCs also showed a 2.82-fold upregulation of the ectodermal marker *Map2* relative to the WT control (Figure 6B). With respect to pluripotency, KO-iPSCs showed downregulation of *Oct4, Nanog, Stella* and *Rex1* compared to WT-iPSCs (Figure 5B). Surprisingly and in contrast to iPSCs, KO-ESCs showed an upregulation of ‘*Oct4*′, which is considered to be the master regulator of the core pluripotency network. Indeed, members of this network like *Sox2, Nanog* and *Stella* are upregulated in KO-ESCs in addition (Figure 6B). Taken together, our data imply that Math6 inhibits the mesodermal fate of pluripotent stem cells.

### 3.8. Inhibition of TGF-β Signalling Restored the Naive Pluripotent State in Math6 KO iPSCs and ESCs

In accordance with our aforementioned link between Math6 and TGF-β signalling in somatic cell reprogramming, we analysed whether Math6 also influences the maintenance of pluripotency by counteracting TGF-β signalling. Both WT and KO, iPSCs and ESCs, were treated with 5 ng/mL TGFb1 for 72 h and analysed for the expression of pluripotency and differentiation markers. As expected, the gene expression data showed an upregulation of the differentiation markers *Fgf5* and *Brachyury* simultaneously to the downregulation of the pluripotency markers *Nanog* and *Stella* in KO-iPSCs. These data clearly show that TGFb1 treatment enriched the primed state in KO compared to WT iPSCs (Figure 6A). With respect to ESCs, TGFb1 treatment resulted in the upregulation of *Oct4* in KO-ESCs compared to WT and KO controls. Subsequently, the other pluripotency markers *Nanog* and *Stella* and the differentiation markers *Fgf5* and *Brachyury* were found to be upregulated. The upregulation of *Oct4* together with *Fgf5* and *Brachyury* in KO-ESCs treated with TGFb1 once again suggests a link between Math6, Oct4 and TGF-β signalling in determining the stem cell fate (Figure 6B). Following this, we also inhibited the TGF-β signalling in iPSCs and ESCs using SB-431542. As anticipated the inhibition of TGF-β signalling restored the naive pluripotent state in KO-iPSCs by downregulating the differentiation markers *Fgf5* and *Brachyury* simultaneously to an upregulation of the pluripotency markers *Stella* and *Nanog* (Figure 6A). Concerning KO-ESCs, the inhibition of TGF-β signalling significantly downregulated *Fgf5* and *Brachyury*, although not entirely (Figure 6B). In contrast to the results achieved for somatic cell reprogramming of WT fibroblasts, we did not observe any significant changes in the mRNA levels of *Math6* in WT-iPSCs and ESCs after treatment of TGFb1 and SB-431542 (Figure 6A,B).

### 3.9. Lack of Math6 Results in the Mesendodermal Specification of Pluripotent Stem Cells

**Figure 7:**
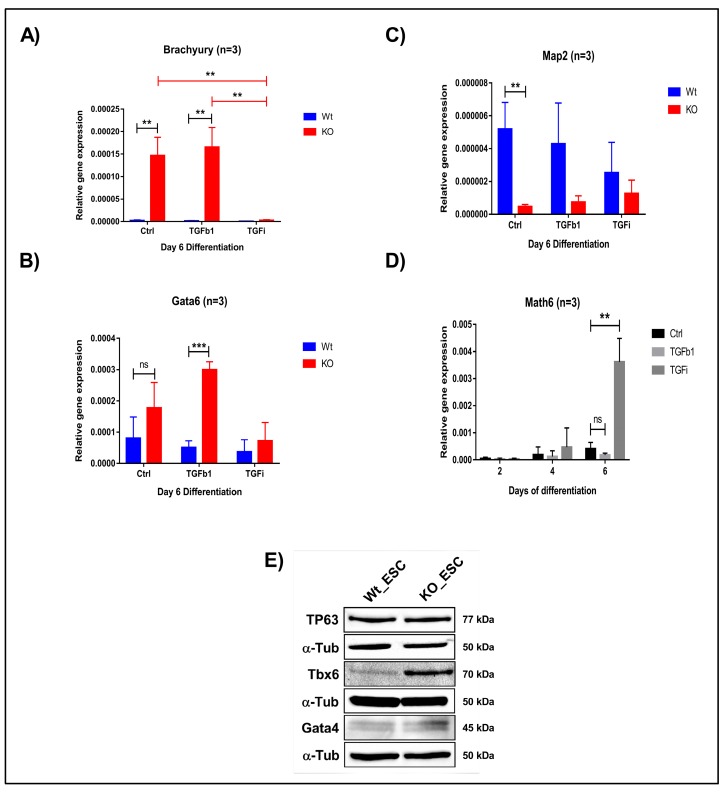
Math6 regulates the stem cell fate by influencing TGF-β signalling. (**A**) Expression of the mesodermal marker *(Brachyury)* during differentiation of WT-ESCs and KO-ESCs on day 6 under the following conditions: Ctrl, TGFb1-treated, SB-431542-treated. The gene expression shown is relative to *18s*. (**B**) Expression of the endodermal marker *(Gata6)* during day 6 of differentiation WT-ESCs and KO-ESCs in following conditions Ctrl, TGFb1 treated, SB-431542 treated. The gene expression shown is normalized to *18s*. (**C**) Expression of the ectodermal marker *(Map2)* during differentiation of WT-ESCs and KO-ESCs on day 6 under the following conditions: Ctrl, TGFb1-treated, SB-431542-treated. The gene expression shown is relative to *18s*. (**D**) Expression of *Math6* during differentiation from day 2-6. The *Math6* expression is inhibited by TGFb1 and TGF-β inhibition resulted in the upregulation of *Math6* expression on day 4 and 6 of differentiation. Significance levels are shown as *(ns) no significance* *p* > 0.05, * *p* ≤ 0.05, ** *p* ≤ 0.01 and *** *p* ≤ 0.001 (**E**) Western blot analysis showing the presence of germ layer markers for ectoderm (TP63), mesoderm (Tbx6) and endoderm (Gata4). α-Tubulin is used as a control.

To further confirm the fate of WT and KO derived iPSCs and ESCs, we withdrew 2i/LIF medium and supplemented the cells with differentiation medium (KOSR). We then analysed the expression of all three germ layer markers on day 2, 4 and 6. We observed the downregulation of ectodermal marker *Map2* and the upregulation of the mesendodermal markers *Brachyury* and *Gata6* (Figure 7A–C and Figure S10). This observation confirms that Math6 acts as a strong regulator of the stem cell fate after pluripotency is lost, that is, it drives stem cells to the ectodermal fate. Subsequently, we also differentiated iPSCs and ESCs in the presence of TGFb1 and SB431542 to validate whether Math6 also influences early differentiation through interfering with TGF-β signalling. As anticipated, TGFb1 treatment augments (*Gata6*) whereas TGF-β inhibition reverted (*Gata6* and *Brachyury*) the mesendodermal fate in KO-iPSCs and ESCs (Figure 7A,B). At the same time, we also observed upregulation in the ectodermal marker *Map2* (Figure 7C). Simultaneously, we observed a strong reverse correlation between the expression of *Math6* and that of the mesendodermal markers *Brachyury* and *Gata6*. As *Math6* expression increases in WT cells under TGF-β inhibition, the expression of *Brachyury* and *Gata6* was found to be downregulated. Again, this data illustrates the interdependent and reverse action of Math6 and TGF-β signalling in the determination of the stem cell fate (Figure 7D).

## 4. Discussion

### 4.1. Math6 in Somatic Cell Reprogramming

Transcription factors of the bHLH family are well known for their ability to orchestrate and regulate crucial genes that are involved in embryogenesis [28]. Like many other bHLH transcription factors, studies performed in chicken, zebrafish and mice have shown that Atoh8 is necessary for multiple developmental events during embryogenesis. Notably, in our previous research, we reported the expression of Math6 in the ICM of mouse blastocysts specifically in the pluripotent ESCs [11]. Moreover, we described the dynamics in the localization of Math6 suggesting its possible role in controlling the pluripotency network [11]. In the current study, we are demonstrating its significance in mesenchymal-epithelial transition during somatic cell reprogramming, maintenance of pluripotency and early differentiation.

The quantification of mRNA levels of *Math6* during reprogramming showed similar expression in all three phases of the reprogramming process. However, Western blot performed to quantify the amount of protein showed an increasing amount of Math6 protein as the cells advance in the reprogramming process. Immunostaining performed at different phases of reprogramming revealed an increase in Math6 protein particularly in the cells which have initiated MET and are committed towards the iPS state. The restricted presence of Math6 protein in the committed reprogramming cells suggests its distinguished role in reprogramming. The lack of correlation between RNA and protein levels of Math6 during reprogramming suggests a possible regulation at the post-transcriptional level.

Our reprogramming experiments performed on feeder cells have revealed the significance of Math6 in the establishment of pluripotency. Reprogramming of Math6 KO fibroblasts on feeder cells demonstrated a disrupted MET compared to WT cells. However, the KO fibroblasts could undergo proper MET and achieve the pluripotent state when cultured in feeder-free conditions albeit with lower reprogramming efficiency. Considering the difference in phenotype between KO cells reprogrammed on the feeder and those under feeder-free conditions, it seems that the feeder cells are impeding the reprogramming process. Feeder cells like mouse embryonic fibroblasts (MEF) are widely used in reprogramming and maintenance of pluripotent cells. Their contribution during reprogramming has not been studied in detail. Mouse embryonic fibroblasts secrete several growth factors including Activin-A and TGF-β, which have been described as potent activators of mesenchymal genes [19,29].

In the early phase of somatic cell reprogramming, cells activate pro-epithelial genes by inhibiting pro-mesenchymal genes [24,27,30]. Our gene expression analysis performed on KO cells during feeder-free reprogramming showed an upregulation of the pro-mesenchymal genes *Smad4, Zeb2, Snail1* and *Snail3* and downregulation of the epithelial marker *E-Cadherin*. Previous studies showed that MET is a prerequisite for the reprogramming and that downregulation of E-Cadherin significantly reduces the reprogramming efficiency [27]. Based on our results and the current literature, we can deduce that Math6 acts as a promoter of MET during reprogramming. Several studies performed on cancer have already shown that bHLH transcription factors in alliance with Snail1 and Zeb repress E-cadherin by promoting the mesenchymal phenotype [31]. Recently, studies performed in cancer cells showed that inhibition of Atoh8 resulted in the downregulation of E-cadherin and upregulation of Vimentin bestowing mesenchymal phenotype to the cells [16]. The phenotype and gene expression data of Math6 KO reprogramming cells are in line with the data published by Wang’s group [16].

In addition to this, we have also observed downregulation of Yamanaka factors along with *Nanog* from day 10 of reprogramming in Math6 KO fibroblasts. The correlation between the downregulation of *E-cadherin*, upregulation of mesenchymal markers during the initiation phase and reduced expression of a pluripotency factor during the intermediate phase corresponds to the observed lower reprogramming efficiency of Math6 KO fibroblasts.

TGF-β signalling is a key regulator of EMT [32] in the way that it has been described to be a potential activator of this process [25]. The treatment of WT and KO reprogramming cells with TGFb1 during the initiation phase severely impaired the reprogramming process in both. At the same time, Math6 was found to be downregulated in the control WT cells. The reprogramming efficiency was also reduced because of TGFb1 treatment during the initiation phase. Accordingly, the inhibition of TGF-β signalling using SB-431542 in KO reprogramming cells led to a reduced expression of mesenchymal markers. In line with this, the reprogramming efficiency in KO cells could be improved by inhibition of TGF-β signalling. At the same time, we observed upregulation in the expression of Math6 in WT reprogramming cells treated with SB-431542. This data is in line with the finding of others [27,30]. In summary, our data show, that a lack of Math6 in combination with TGF-β acts synergistically, whereas Math6 and TGF-β act antagonistically on MET and somatic cell reprogramming: Math6 promotes MET and reprogramming.

### 4.2. Math6 in the Maintenance of Pluripotency and Early Differentiation

We have previously reported the presence of Math6 in pluripotent ESCs during embryogenesis [11]. The present study shows that the lack of Math6 leads to priming of pluripotent stem cells towards the mesendodermal fate. Furthermore, we show an upregulation of *Oct4* in Math6 KO-ESCs. It has been reported that the dosage of Oct4 is critical to define the fate of pluripotent stem cells. A slight increase or decrease in the expression of Oct4 in pluripotent stem cells results in the specification of mesendodermal and trophectodermal lineage, respectively [33]. The downregulation of Oct4 was reported in the epiblast in colocalization with the TGF-β related factor nodal [34], as well as in Smad2 deficient mice [35], thus linking Oct4 expression to TGF-β signalling, that is, TGF-β represses Oct4 expression. The absence of mesoderm was also observed in Smad3 deficient mice [36] and Smad4 deficient mice [37]. Evidently, another study performed on mouse ESCs showed that a transient increase in the expression of Oct4 is necessary for the TGF-β induced Smad-mediated mesoderm specification [38]. In contrast to this, we did not observe any significant changes in the mRNA levels of *Oct4* in WT-ESCs and KO-ESCs treated with TGFb1 and SB-431542. This could be because of 72-h prolonged treatment of ESCs in serum-free 2i/LIF culture conditions which might have stabilized *Oct4* levels. Compared to WT-ESCs, the treatment of KO-ESCs with TGFb1 significantly increased the expression of the mesodermal marker *Brachyury* and subsequently, inhibition of TGF-β signalling showed downregulation of *Brachyury*. At the same time, the other pluripotency markers *Sox2* and *Nanog* were also found to be upregulated in KO-ESCs. Sox2 upregulation was shown to prime ESCs towards neuroectoderm [39]. The upregulation of *Map2* in KO-ESCs corresponds to this. However, we did not observe any upregulation of *Map2* during differentiation. Nanog is downstream to Oct4 and it has been reported that upregulation of Nanog also results in the mesendodermal specification of pluripotent stem cells [40] which might explain the downregulation of *Map2* in the differentiation phase. The upregulation of *Oct4* and *Nanog* in KO-ESCs and commitment of KO-ESCs towards mesendodermal fate clearly indicates the link between Oct4, TGF-β and Math6.

Confirming the involvement of Math6 in counteracting TGF-β signalling, KO-ESCs treated with TGFb1 during differentiation showed an increase in the expression of mesendodermal markers *Brachyury* and *Gata6*. At the same time, the inhibition of TGF-β signalling during differentiation rescued the mesendodermal specification in KO-ESCs. At this point, comparing the differentiation of WT-ESCs with KO-ESCs revealed a strong correlation between the expression of Math6 and germ layer markers. The upregulation of *Math6* with TGF-β inhibition inversely correlates with the downregulation of *Brachyury* and *Gata6*. Similarly, the downregulation of *Math6* with TGFb1 treatment inversely correlates with the upregulation of *Brachyury* and *Gata6*. Based on these data it is tempting to speculate that Math6 acts upstream to Oct4 and downstream to TGF-β signalling. Supporting this, a study performed in hepatocellular carcinomas proposed Math6 as a repressor of Oct4 and Nanog [15]. Taken together, we describe Math6 as a novel regulator of MET, a regulator of stem cell fate and could demonstrate that it counteracts TGF-β signalling.

## Supplementary Materials

The Supplementary information 1 & 2 are available online at https://www.mdpi.com/2073-4409/8/6/529/s1.
