# Supplementary material for: bHLH Transcription Factor Math6 Antagonizes TGF-β Signalling in Reprogramming, Pluripotency and Early Cell Fate Decisions
Source: Cells. 2019 Jun 2;8(6):529. doi: 10.3390/cells8060529 (PMC6627693; doi:10.3390/cells8060529)
Supplement: Supplementary file 1 [file cells-08-00529-s001.zip › supplementary/Supplementary information_1.docx]

# **Supplementary information: 1**


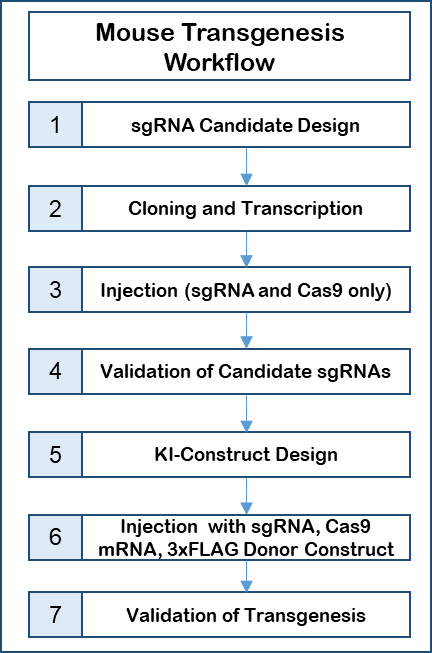


**Figure S1:** Schematic of CRISPR-Cas9 workflow.


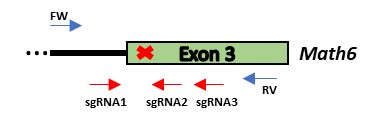


Figure S2: Schematic representation of mMath6 targeting region. Candidate crRNA sequences (red arrows, directionality towards PAMs) targeting the proximity of the last mMath6 coding triplet, stop codon (in red) and 5‘UTR were chosen for sgRNA template design. Blue arrows: Forward (FW) and reverse (RV) primers amplification of the targeting region (793 bp).

**Target region genotyping primers:**

FW: 5’-CCTTGGAGGTGTGACTCTGG-3’

RV: 5’-TTGTCGTGGCTCTAGGAAGC-3’

**Math6 crRNA sequences:**

SgRNA1: 5’- TCTGTGGTCTAGCCTGTGGCAGG-3’

- guide #13 quality score: 61 guide sequence: TCTGTGGTCTAGCCTGTGGCAGG on-target locus: chr6:-72157080 number of offtarget sites: 273 (30 are in genes) top 20 genome-wide off-target sitesshow all exonic sequence score mismatches UCSC gene locus TAACTGGTCTAGCCTGTGGCCAG 2.3 3MMs [2:3:4] NM_172122 chr4:+140575335 TGTTTGGTCTAGCCTGTGGTTGG 1.5 3MMs [2:4:20] chr2:-166452202 TGGGTGGTCTAGCCTGTGGGAGG 1.5 3MMs [2:3:20] chr19:+58982330 TCAGTGGTTAAGCCTGTGGCTAG 1.4 3MMs [3:9:10] chr7:-17380441 CCCCTGGGCTAGCCTGTGGCAGG 1.4 4MMs [1:3:4:8] chr14:+58766633 ACGGTGGCCGAGCCTGTGGCTGG 1.3 4MMs [1:3:8:10] chr2:-166030422 TCTCAGGGCCAGCCTGTGGCAGG 1.3 4MMs [4:5:8:10] chr2:+28381332 TGTCTGGTCTAGCCTGAGGCAAG 1.3 3MMs [2:4:17] chr8:-92701449 TATCTGGTCTAGTCTGTGGCAAG 1.1 3MMs [2:4:13] chr3:+142685270 TCTGGGATGTAGCCTGTGGCAGG 1.0 3MMs [5:7:9] chr10:-51273653 TCTGTATCCTAGCCTGTGGCTGG 1.0 3MMs [6:7:8] NM_199068 chr5:-142933167 CCTGTGGTGTAACCTGTGGCAGG 0.9 3MMs [1:9:12] chr9:+43209143 TCTCTGTGCAAGCCTGTGGCTGG 0.9 4MMs [4:7:8:10] chr4:-99994503 CCTGGGGTCTAGCCGGTGGCAGG 0.8 3MMs [1:5:15] chr15:-75908732 CCTGGGGTTAAGCCTGTGGCTAG 0.8 4MMs [1:5:9:10] chr10:+79344099 TCAGGTGTCCAGCCTGTGGCCAG 0.8 4MMs [3:5:6:10] chr9:-68854635 ACTATGGTCAGGCCTGTGGCAGG 0.7 4MMs [1:4:10:11] chr2:+180671726 TGTCTGGTCAGGCCTGTGGCAGG 0.7 4MMs [2:4:10:11] chr9:+117351906 GGTGTGGCCTAGCCTGTGGGTAG 0.7 4MMs [1:2:8:20] chr14:+35861343 TCTCTGGGCATGCCTGTGGCAAG 0.7 4MMs [4:8:10:11] chr16:-7943697 AGAGTGGTCTAGCCTGTGGAGGG 0.7 4MMs [1:2:3:20] chr1:+184805133 TGTCTGGGCTAGCCTGTGGAAGG 0.7 4MMs [2:4:8:20] chr7:+146727673 TCTGTGGTCTAGCCTGTGCAAGG 0.7 2MMs [19:20] chr2:+126773923 TGTTTGGTCAAACCTGTGGCCAG 0.7 4MMs [2:4:10:12] chr1:+57683255 GCAGTGGTCCAGCCTGTGGAAAG 0.6 4MMs [1:3:10:20] NM_145920 chr5:+37774246 TTTCTGGTCAAGGCTGTGGCCAG 0.5 4MMs [2:4:10:13] NM_133962 chr8:-3455049 TCTTAGATCTGGCCTGTGGCTGG 0.5 4MMs [4:5:7:11] chr2:+152483211 TCTCTGGTCTATACTGTGGCTGG 0.5 3MMs [4:12:13] chr10:+23487590 TCTCTCCTCCAGCCTGTGGCTGG 0.5 4MMs [4:6:7:10] chr1:+40194437 TCTGAGATAAAGCCTGTGGCTGG 0.5 4MMs [5:7:9:10] chr15:+87772216 TCTTCGGGCTAGCCTGTGCCTGG 0.5 4MMs [4:5:8:19] NM_173427 chr4:+139519560 CCTGTTGTTGAGCCTGTGGCAGG 0.5 4MMs [1:6:9:10] chr6:-147650369 TGTGGGGTCCAGCCTGTGACTGG 0.5 4MMs [2:5:10:19] NM_001081158 chr11:-74473605 TTCGTTGTCTTGCCTGTGGCTAG 0.5 4MMs [2:3:6:11] NM_153179 chr1:-20063640 TCTTCTGTCTGGCCTGTGGCAGG 0.5 4MMs [4:5:6:11] chr11:+62068361 TCTTTTGTGGAGCCTGTGGCGGG 0.5 4MMs [4:6:9:10] chr16:-4434890 TCTGCAGTGAAGCCTGTGGCCAG 0.5 4MMs [5:6:9:10] chr15:+32271897 TCTATGTTCCAGCCTGTGGTGAG 0.4 4MMs [4:7:10:20] chr2:+160950327 TCTGTGGTCAAGGCTGTGGACAG 0.4 3MMs [10:13:20] chr10:-83943984 TCTGTGGTCCAGCCTGAGGTGAG 0.4 3MMs [10:17:20] NM_206973 chr19:+4143546 GCTGTGGTTCATCCTGTGGCTGG 0.4 4MMs [1:9:10:12] chr16:+42120885 TCTGTGGATGGGCCTGTGGCTAG 0.4 4MMs [8:9:10:11] chr1:+14086874 CCTGTGGACTGTCCTGTGGCTGG 0.4 4MMs [1:8:11:12] chr3:-8633275 ACAGTGGTCTTTCCTGTGGCAAG 0.4 4MMs [1:3:11:12] chr2:+71734290 ACTGTGGACTGGCCTGTGGTGAG 0.4 4MMs [1:8:11:20] chr10:-84117839 TGTGTGGGCTCGCCTGTGGGTAG 0.4 4MMs [2:8:11:20] chr1:+89482830 TCCCTGGTCTTCCCTGTGGCAGG 0.4 4MMs [3:4:11:12] chr7:+120225926 TCAGTGGCCTGACCTGTGGCCAG 0.4 4MMs [3:8:11:12] chr1:+133863127 TCTGAGGTCAAGCATGTGGCTAG 0.4 3MMs [5:10:14]

SgRNA2: 5’- GAATGAGGCTGGTCGTCTCCTGG-3’

- guide #1 quality score: 84 guide sequence: GAATGAGGCTGGTCGTCTCCTGG on-target locus: chr6:-72157153 number of offtarget sites: 121 (16 are in genes) top 20 genome-wide off-target sitesshow all exonic sequence score mismatches UCSC gene locus GCAGGAGCCTGGTCGTCTCCGGG 2.5 3MMs [2:4:8] NM_001033382 chr6:-119299150 CTATGAGGGGGGTCGTCTCCTAG 0.8 4MMs [1:2:9:10] chr5:+140651773 GATCAAGGCTGGTCGTCTCACAG 0.7 4MMs [3:4:5:20] chr2:-174483445 GCAAGAGGCAGATCGTCTCCAGG 0.7 4MMs [2:4:10:12] chr12:+25067703 AAGTGTCGCTGGTCGTCTCCCAG 0.6 4MMs [1:3:6:7] chr4:-109656644 GAATTTGGAAGGTCGTCTCCTGG 0.5 4MMs [5:6:9:10] chr15:+67252776 CAATGAGGCAGGTGGTCTCCAGG 0.4 3MMs [1:10:14] chr16:+18458413 GAGTCAGGTTGGTCGTCTCAGGG 0.4 4MMs [3:5:9:20] chr18:-74282240 AACTGAGTCTGGTCTTCTCCTAG 0.4 4MMs [1:3:8:15] chr7:+88434755 GTAAGCGGCTGGTCGTTTCCTGG 0.4 4MMs [2:4:6:17] NM_138674 chr15:-44388470 GGTTGTGGCTGGTCGTGTCCTAG 0.4 4MMs [2:3:6:17] NM_028195 chr15:-78448900 GCATGAAGCTGGTAGTCTCCTGG 0.3 3MMs [2:7:14] chr6:+127362287 GAATCAGGCTGGCCTTCTCCAAG 0.3 3MMs [5:13:15] chr10:-59598064 TGATGACGCTGGTCTTCTCCTGG 0.3 4MMs [1:2:7:15] chr8:-117864981 GAATGAGTTTGGTCGACTCCAGG 0.3 3MMs [8:9:16] chr1:+69422535 CAATGAGACTGGTCGTTTCATGG 0.3 4MMs [1:8:17:20] chr1:+132179136 CACTGAGGCTGCTCGTCTGCTAG 0.3 4MMs [1:3:12:19] chr17:-72919349 AAATGAGTATGGTCATCTCCTGG 0.3 4MMs [1:8:9:15] chr2:-104179017 CAGTGTGGCTGGTCCTCTCCTAG 0.2 4MMs [1:3:6:15] chr10:-57110915 GAATGAGACTGGTCGTCCCTGAG 0.2 3MMs [8:18:20] chr3:-41390937 GAGTGGGTCTGGTCCTCTCCAGG 0.2 4MMs [3:6:8:15] chr5:+143595201 TGATCAGGCTGGTTGTCTCCCAG 0.2 4MMs [1:2:5:14] chr18:-82120392 CACTGAGCCTGGTGGTCTCCAAG 0.2 4MMs [1:3:8:14] chr5:+121760409 GGCAGAGGCTGGTGGTCTCCAGG 0.2 4MMs [2:3:4:14] NM_001256081 chr7:-105223444 GATTTAGACTGGTGGTCTCCAGG 0.2 4MMs [3:5:8:14] chr4:+133567617 GAATGAGGCAGGTCATCTACAGG 0.2 3MMs [10:15:19] chr4:+54355540 GAATGAGACTGGTCGTGACCTGG 0.2 3MMs [8:17:18] chr8:+114511361 GAATGTGTATGGTCGTTTCCCAG 0.2 4MMs [6:8:9:17] chr10:+58865603 GATTGAGGCTGCTTGTCTCCCAG 0.2 3MMs [3:12:14] chr3:-153486253 AAATGAGCCTGCTCATCTCCCAG 0.2 4MMs [1:8:12:15] chr6:+113936945 AAAGGAGGCTGTTCTTCTCCAGG 0.2 4MMs [1:4:12:15] chr13:+6921447 GAATGAGGATGCTCTTCTCCCAG 0.2 3MMs [9:12:15] chr11:+20712341 GAAGGAGACGGGTGGTCTCCTGG 0.2 4MMs [4:8:10:14] chr6:+35266001 GAATGTGAGTGGCCGTCTCCTAG 0.2 4MMs [6:8:9:13] chr12:+111999963 TGATGAGGCTGGTCTTCTCAAGG 0.2 4MMs [1:2:15:20] NM_138748 chr2:+30303419 TGATGAGGCTGGTCTTCTCAAGG 0.2 4MMs [1:2:15:20] NM_138748 chr2:+30302469 CACTGAGGCTGGTCATCTCACAG 0.2 4MMs [1:3:15:20] chr17:-57031606 GTATGAGGCTGGTAGTTTCCAAG 0.2 3MMs [2:14:17] chr14:+102978283 GAAGGAGACTGGTCATGTCCAAG 0.2 4MMs [4:8:15:17] chr16:+17214426 GGTTGAGGCTGGCCCTCTCCCAG 0.2 4MMs [2:3:13:15] chr7:-126236090 CTATGATGCTGGTGGTCTCCAGG 0.2 4MMs [1:2:7:14] chr7:+17663634 GTCTGAGGCTAGTCGGCTCCGGG 0.1 4MMs [2:3:11:16] chr13:+55937366 GCATGTGGCTGGCCGTGTCCAGG 0.1 4MMs [2:6:13:17] chr7:-71890961 GCATCAGGTTGGTAGTCTCCTAG 0.1 4MMs [2:5:9:14] chr17:+89247046 GAATGAAGCATGTCATCTCCCAG 0.1 4MMs [7:10:11:15] chr12:-16212116 GAGAGGGGCTGGTGGTCTCCCGG 0.1 4MMs [3:4:6:14] NM_001039692 chr18:+6136018 GAATGAGGTTTGTGGTCTCCCAG 0.1 3MMs [9:11:14] chr11:+88497511 GAATGTGGCTGTTTGTCTCCTGG 0.1 3MMs [6:12:14] chr2:+89139391 GAATGAGGGAGGGCGTGTCCCAG 0.1 4MMs [9:10:13:17] NM_008037

SgRNA3: 5’- GAAGTTGGATGCTAACACTC-3’

- guide #6 quality score: 71 guide sequence: GAGTGTTAGCATCCAACTTCAGG on-target locus: chr6:+72157014 number of offtarget sites: 144 (12 are in genes) top 20 genome-wide off-target sitesshow all exonic sequence score mismatches UCSC gene locus GAGGGTTAGAATCCAACTTCTAG 6.2 2MMs [4:10] chr1:-71149846 GAGTGTCAGCATCCAACTTTCAG 3.1 2MMs [7:20] chrX:-33876019 GAATTTTAGCATCCAACTTTAAG 1.4 3MMs [3:5:20] chr4:+4635405 CTTGGTTAGCATCCAACTTCCGG 1.3 4MMs [1:2:3:4] chr3:+52314273 CCCTGTCAGCATCCAACTTCTGG 0.9 4MMs [1:2:3:7] chr5:-134713229 ATGTGTGAGAATCCAACTTCTAG 0.9 4MMs [1:2:7:10] chr19:-31447422 TCGTGCTGGCATCCAACTTCTGG 0.8 4MMs [1:2:6:8] chr3:-123206934 GCCTGTTAGCATCCTACTTCCAG 0.8 3MMs [2:3:15] chrX:+54124965 AATTCCTAGCATCCAACTTCCAG 0.8 4MMs [1:3:5:6] chr10:+128331934 CATTCTTAGCTTCCAACTTCCAG 0.8 4MMs [1:3:5:11] NM_001104927 chr4:-96137138 GCATCTTAGCCTCCAACTTCAAG 0.8 4MMs [2:3:5:11] chr13:-47430655 CAGTGTTAGCACCCAACTTGCAG 0.8 3MMs [1:12:20] chr8:-119594650 GGATGTTGGCATCCAACTTAGGG 0.7 4MMs [2:3:8:20] chr14:-25660521 CAGTGTCTTCATCCAACTTCAGG 0.6 4MMs [1:7:8:9] chr6:-77017099 AAATGTTTGCATACAACTTCAGG 0.6 4MMs [1:3:8:13] chr2:+55970107 GATTAAGAGCATCCAACTTCCAG 0.5 4MMs [3:5:6:7] chr14:+12436776 GAGTGTCAACATCCAACTTGCAG 0.5 3MMs [7:9:20] chr2:+3737309 CAATGTGAGCACCCAACTTCAAG 0.5 4MMs [1:3:7:12] chr14:+11321333 GTGCGTTACCTTCCAACTTCTAG 0.5 4MMs [2:4:9:11] chr1:-91625319 GAGTTTCAGAGTCCAACTTCAGG 0.5 4MMs [5:7:10:11] chr13:+6021809 GGCTGTGAGCATCCAACTTATAG 0.5 4MMs [2:3:7:20] chr10:+17244267 GAGTCCTGGCCTCCAACTTCTAG 0.5 4MMs [5:6:8:11] chr5:+37379932 GTGTGTTTCCATCCAACTTTTGG 0.4 4MMs [2:8:9:20] chr17:-71308345 GAATTTTAGCTTCCAACTTGAAG 0.4 4MMs [3:5:11:20] chr9:-56262473 GACTATTAGCCTCCAACTTGTAG 0.4 4MMs [3:5:11:20] NM_001080925 chr11:-98712482 AAGTGTTAGGCACCAACTTCAGG 0.4 4MMs [1:10:11:12] chr6:-27545191 TATTGATAGCATCCAATTTCTGG 0.4 4MMs [1:3:6:17] chr2:+86136945 GAATGCTGGCATCCAAATTCAGG 0.4 4MMs [3:6:8:17] chr6:+107032367 GGGAGTTAACATGCAACTTCGAG 0.3 4MMs [2:4:9:13] chr5:+44400515 AGGTGTTTGCATCCAACCTCAGG 0.3 4MMs [1:2:8:18] chr17:-14370401 GACACTTAGCATCCAACATCAAG 0.3 4MMs [3:4:5:18] chr15:-7818469 GTGTGCCAGCATCCAACTTTAGG 0.3 4MMs [2:6:7:20] chr2:-13399485 GACTGTTAAAATCCAACTGCTAG 0.3 4MMs [3:9:10:19] chr10:-63647077 GAGTGTGCACACCCAACTTCTAG 0.3 4MMs [7:8:9:12] chr5:+138649012 CAGTGTGAGCCTCCAACTTTTAG 0.3 4MMs [1:7:11:20] chr7:+36175559 GAGTCTTAGTACCCAAGTTCAGG 0.3 4MMs [5:10:12:17] chr14:+67778643 GATTCTTGGCATCCATCTTCTGG 0.3 4MMs [3:5:8:16] chr15:-40206185 GAGTGACAGTAACCAACTTCTGG 0.3 4MMs [6:7:10:12] chr14:+60306836 GAGTGATAATTTCCAACTTCAAG 0.3 4MMs [6:9:10:11] chr16:+78321613 TAGTATTAGTATCCATCTTCCAG 0.3 4MMs [1:5:10:16] chr13:-55398956 GAATGTTGGCAGCCAACTGCAGG 0.2 4MMs [3:8:12:19] chr2:-61953552 GAGGGAGAGCATCCAAATTCTAG 0.2 4MMs [4:6:7:17] chr16:-62874325 GAGGATTAGCATTCAATTTCTGG 0.2 4MMs [4:5:13:17] chrX:-109809475 GACTGTCAGCAACCAACTTTGAG 0.2 4MMs [3:7:12:20] NM_001024478 chr12:-33770851 CAGTGTTTGCATCCAACTCTGAG 0.2 4MMs [1:8:19:20] chr1:-155112392 TATTGTTAGCATCCAACTGAAAG 0.2 4MMs [1:3:19:20] chr4:+13506086 GATTGTTAGCATCTAACTTACAG 0.2 3MMs [3:14:20] chr14:+102502097 GAGTGCTAGGTGCCAACTTCTGG 0.2 4MMs [6:10:11:12] chrX:+9630793 GAGGGTTAGCATCCACTTTCTAG 0.2 3MMs [4:16:17]


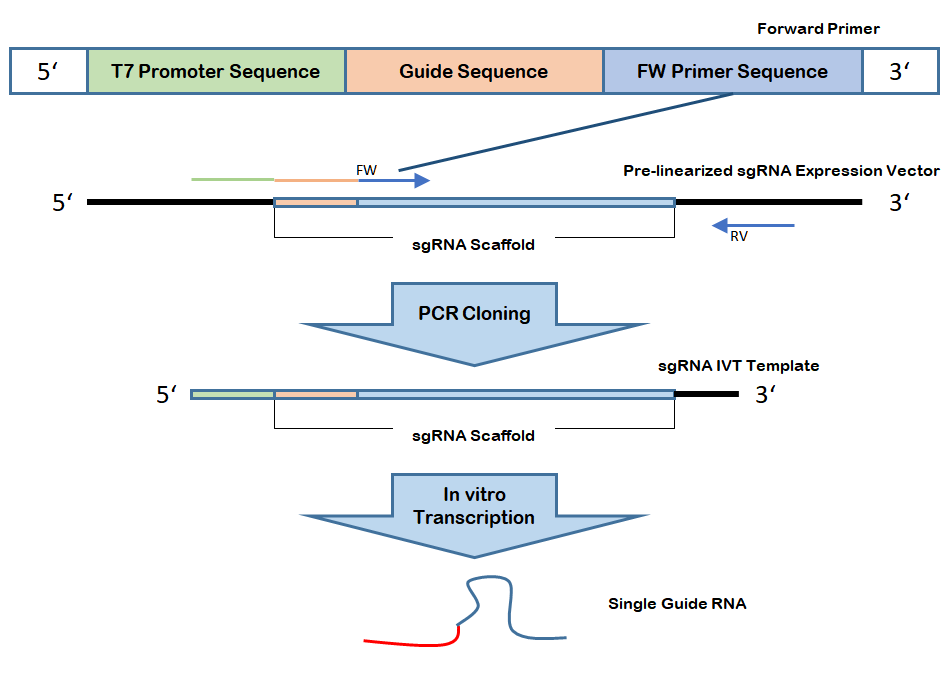


Figure S3: sgRNA template and sgRNA production. Templates for in vitro transcription were produced in a PCR cloning reaction, replacing the current crRNA sequence of the pSLQ1651-sgTelomere (F+E) sgRNA scaffold with Math6 targeting crRNA sequences fused to an upstream T7 promoter. Products from PCR cloning served as IVT templates to produce single-guide RNA.

**T7 promoter-sgRNA-FW:**

Math6 sg1: 5’-TAATACGACTCACTATAGG-TCTGTGGTCTAGCCTGTGGC-GTTTAAGAGCTATGCTGGAA-3’

Math6 sg2: 5’-TAATACGACTCACTATAGG-GAATGAGGCTGGTCGTCTCC-GTTTAAGAGCTATGCTGGAA-3’

Math6 sg3: 5’-TAATACGACTCACTATAGG-GAGTGTTAGCATCCAACTTC-GTTTAAGAGCTATGCTGGAA-3’

RV: 5-tgcatggcggtaatacggttatc-3’

pSLQ1651-sgTelomere(F+E): Bo Huang and Stanley Qi labs (Addgene plasmid #51024)

Figure S4: Micromanipulation of Zygotes that were positioned with a holding needle (to the left) and injected with a piezo-controlled injection needle (to the right). CP: Cytoplasm. PM: Plasma membrane. ZP: Zona Pellucida.

Injection mix composition for sgRNA validation:


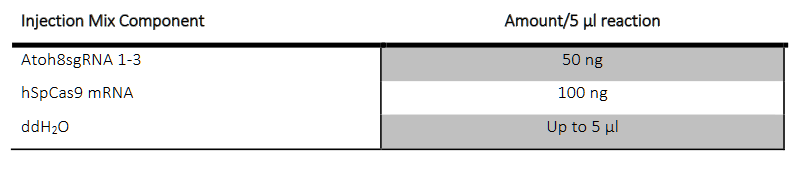


Injection mix for composition for knock-in:


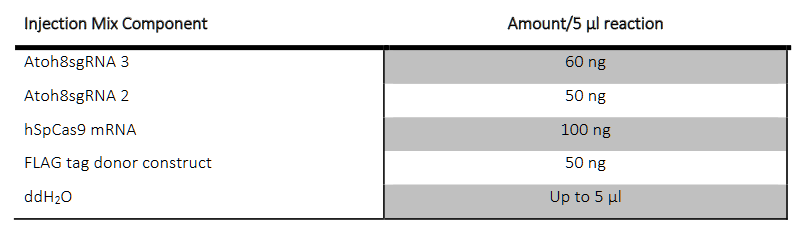


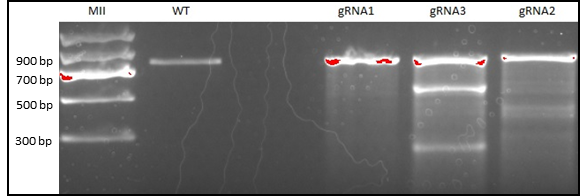


Figure S5: 2 % Agarose gel showing T7 endonuclease reaction products for sgRNA/WT DNA mixtures compared to WT DNA. sgRNA3 displays highest efficiency in form of two fragments of expected sizes that result from genotyping primer qPCR products that were cut by T7 endonuclease as a consequence of mismatch formation between WT and sgRNA3 targeted DNA. sgRNA2 also shows weak bands.


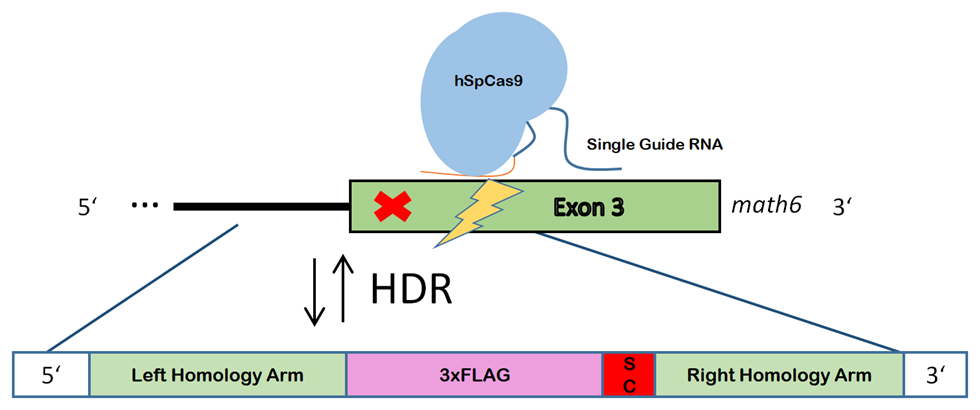


**Figure S6:** Schematic of donor construct design and integration site. HDR: Homology directed repair.

**Donor construct sequence:**

GGGGTTCGGTATCCTCAAGGCAGCAGAATTCAGTGGGGAGAAAGAGGACAGTGTATGCTATGGGGATTGCTAGTCTTGGGTCTAAATCTGGCCCTATTGTATGACTGAAACATTGCCTGGGCAATATAAATCTAATGTCCCATTGTGGGCAGCCATGACAATTGAGAGAATGTCCTGTGAGTGTTAGCATCCAACTTCACCAGGAACAGGAATAATGCTGATATACTTTTCTTTTCAGGAGGATTACAAGGATGACGACGATAAGGACTATAAGGACGATGATGACAAGGACTACAAAGATGATGACGATAAA**TGA**CCTGCCACAGGCTAGACCACAGACACCACTGTGAGTCTCTTCCCAGTTGGGACTGAGGAGAAAGCTGGGACCACCAGGAGACGACCAGCCTCATTCCTTGTCTTCCTCAAGATGCTGCCAGATACTCAGCCCGTGTACCATCTCTCAGGGTCACTTAGAACCAGCACAAGGGCCGCCGGCCTCCCTCTCCG


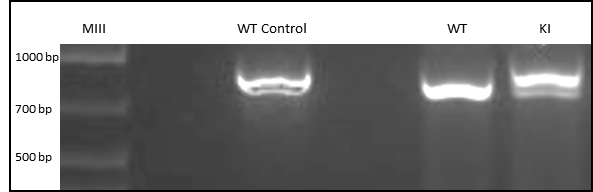


**Figure S7:** 1.5 % Agarose gel showing amplification products of genotyping primers from genotyped mice (right side) in comparison to WT.

**DNA sequence KI mouse as determined by sequencing:**

NNNGNNNANNNATCTTCGGATGGCTCGAGTTTTTCAGCAAGATCCTTGGAGGTGTGACTCTGGAGCTCATGATATCAGCACCAGGTGTCCCTGGGATTAAAGGGGTTCGGTATCCTCAAGGCAGCAGAATTCAGTGGGGAGAAAGAGGACAGTGTATGCTATGGGGATTGCTAGTCTTGGGTCTAAATCTGGCCCTATTGTATGACTGAAACATTGCCTGGGCAATATAAATCTAATGTCCCATTGTGGGCAGCCATGACAATTGAGAGAATGTCCTGTGAGTGTTAGCATCCAACTTCAGGAGGAACAGGAATAATGCTGATATACTTTTCTTTTCAGGAGGATTACAAGGATGACGACGATAAGGACTATAAGGACGATGATGACAAGGACTACAAAGATGATGACGATAAATGACCTGCCACAGGCTAGACCACAGACACCACTGTGAGTCTCTTCCCAGTTGGGACTGAGGAGAAAGCTGGGACCACCAGGAGACGACCAGCCTCATTCCTTGTCTTCCTCAAGATGCTGCCAGATACTCAGCCCGTGTACCATCTCTCAGGGTCACTTAGAACCAGCACAAGGGCCGCCGGCCTCCCTCTCCGTCTCCTGCCATCCAGGGTTACTTCAGATTTTGCCTTCTGCCTGGTGGGGCGGGCTATTGCCAAAGATTCTACAGAGATCGTCCAAGGAAATTATGGATGAGGCCCCAACAGCACAAAACCACATCATTGCTGCCCTACCAAGTCCACCTCAAGCCAAAGATGGATTAGCACTACTGCTAGGAAGCACTTAACCCTCCAAGCCAGTGCTCGCTCTGTGCTGGGCCCTGAGCAGCTGGTTGTCTTCGAGACAAGCTTCCTAGAGCCACGACAAATCTTTCTAGAAGATCTCCTACAATATTCTCAGCTGCCATGGAAAATCGATGTTCTTCTTTTATTCTCTCAAGATTTTCAGGCTGTATATTAAAACTTATATTAAGAACTATGCTAACCACCTCATCANNACCGTTGTAGTGGCGTGGGTTTTCTTGGCAATCGACTCTCATGAAAACTACGAGCTAAATATTCATATNTCNNNGACNACTTTANNCTGCATTTTTTTTTGACGAGTNNNNGCAGCCTCNGNACTGANACNGGNNNTTATTAAAANTAATTTNGAAANAAAGTTCCAGGNNTANTANC
