# Supplementary material for: bHLH Transcription Factor Math6 Antagonizes TGF-β Signalling in Reprogramming, Pluripotency and Early Cell Fate Decisions
Source: Cells. 2019 Jun 2;8(6):529. doi: 10.3390/cells8060529 (PMC6627693; doi:10.3390/cells8060529)
Supplement: Supplementary file 1 [file cells-08-00529-s001.zip › supplementary/Supplementary information_2.docx]

# **Supplementary information 2**


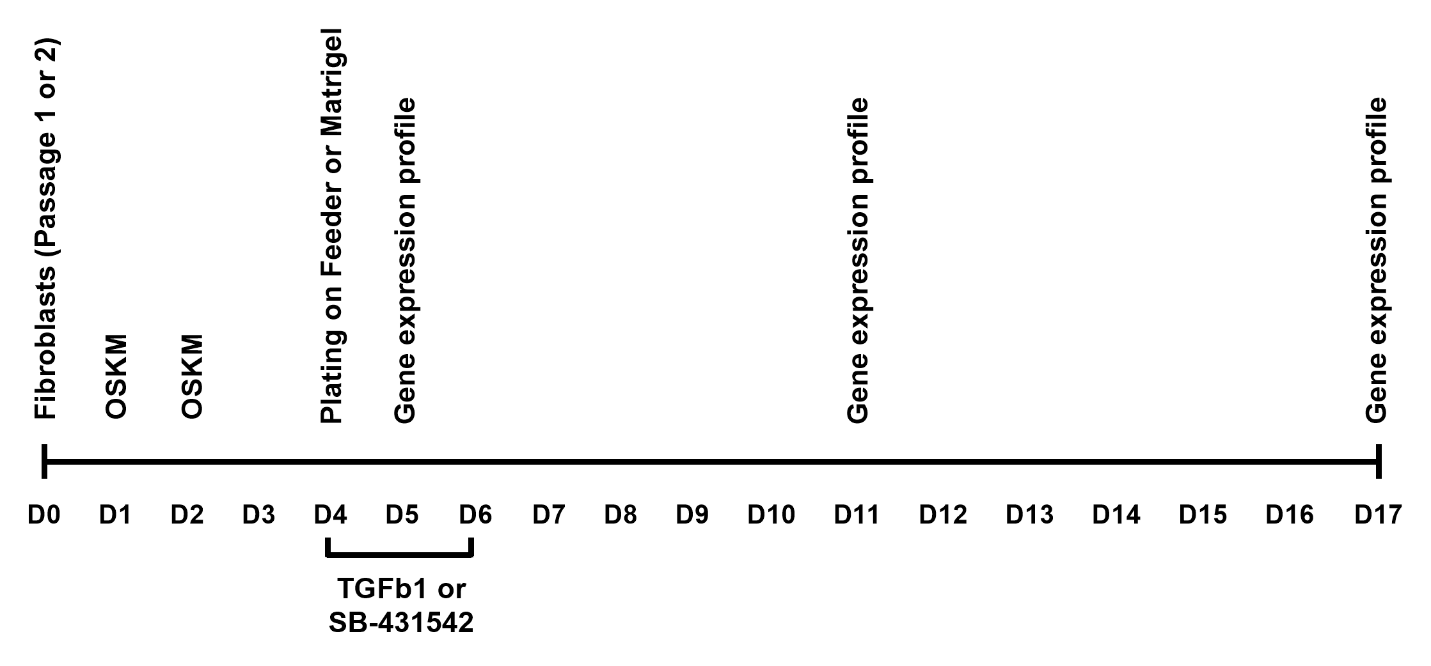


**Figure S8:** Timeline of reprogramming performed under the feeder and feeder-free conditions.


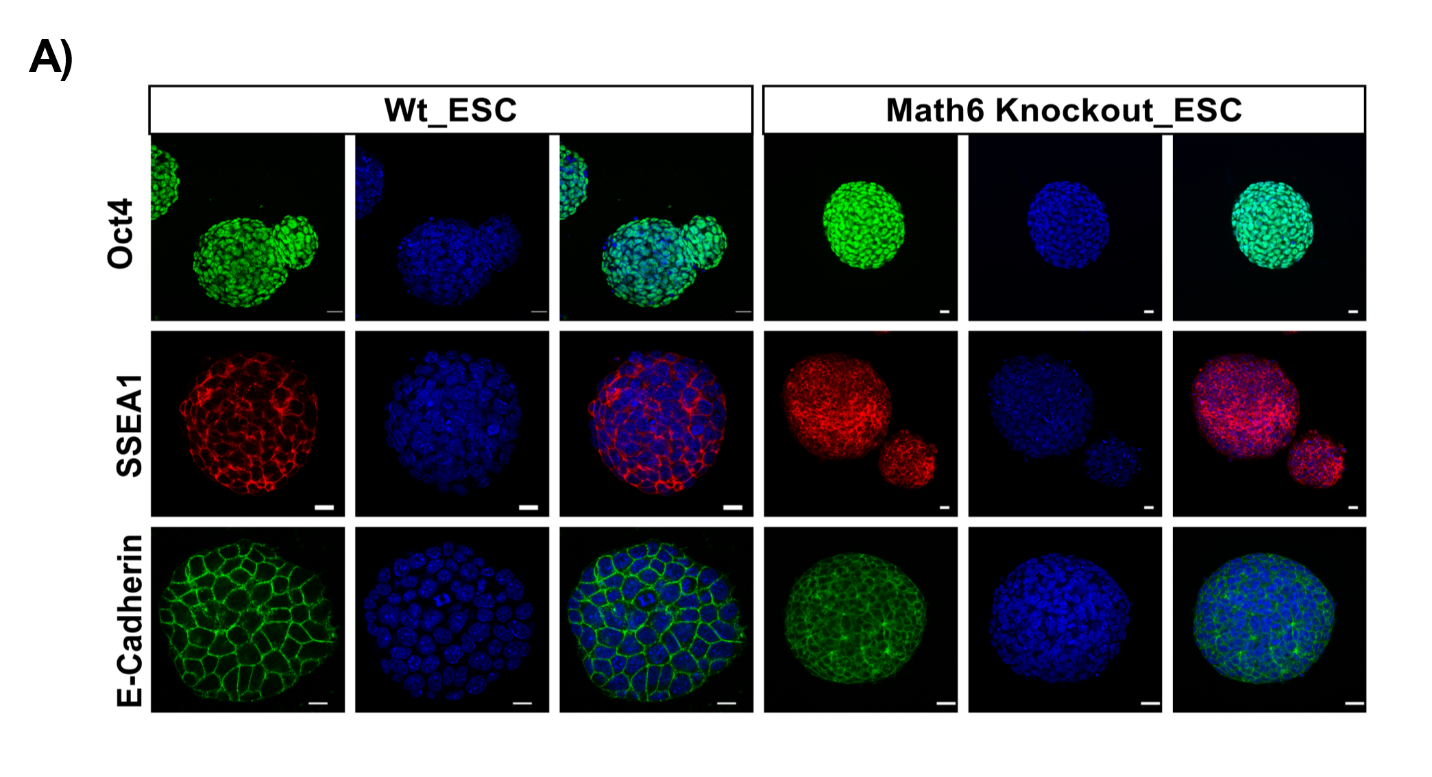


**Figure S9:** WT and KO ESCs stained positive for Oct4 (green), SSEA (red), E-Cadherin (green) and DAPI (blue). Scale bar indicates 20µm.


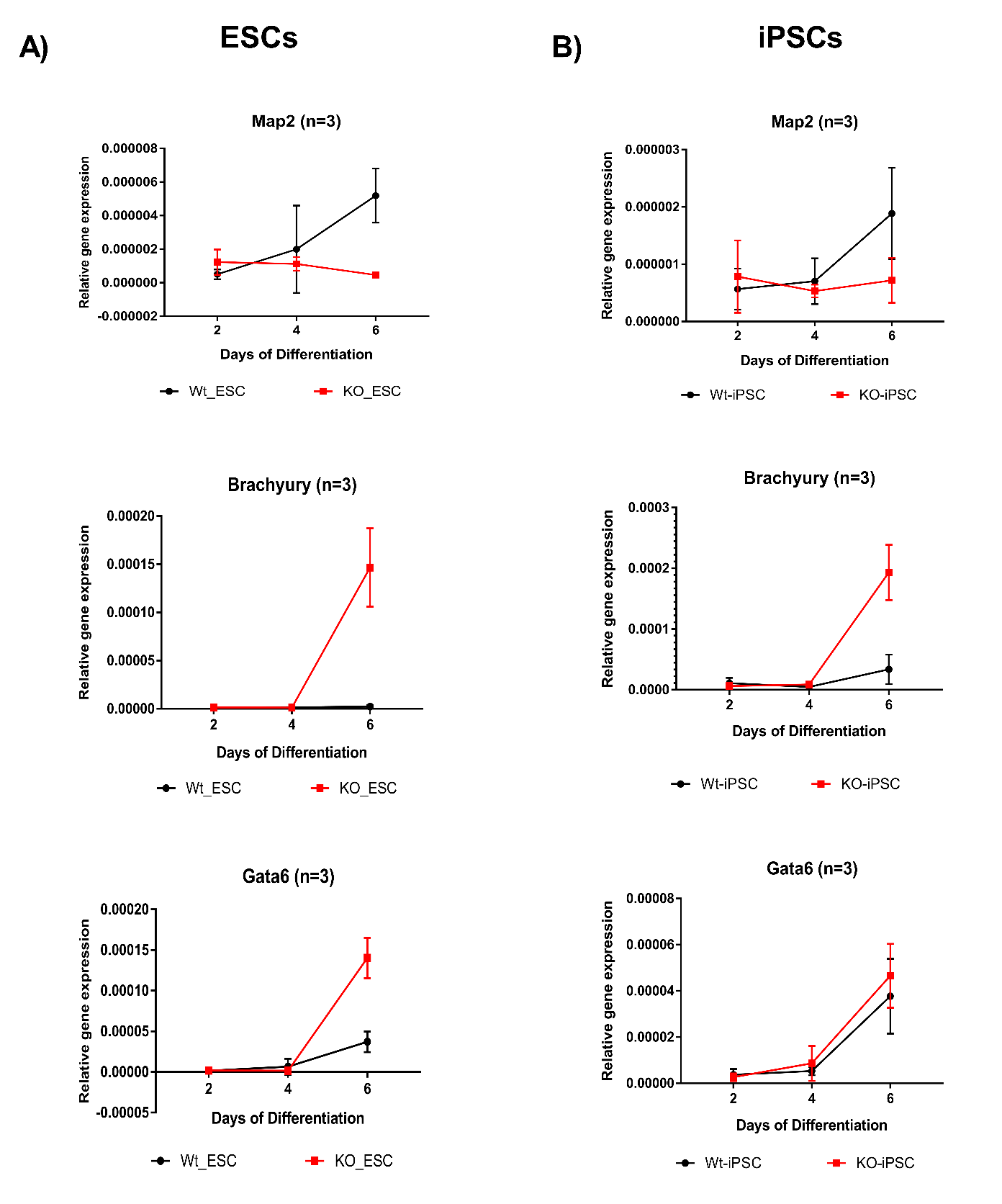


**Figure S10:** A) Expression of ectodermal marker (*Map2*), mesodermal marker (*Brachyury*) and endodermal marker (*Gata6*) during day 2, 4 and 6 of differentiation in Wt and KO ESCs. The gene expression shown is relative to 18s. B) Expression of ectodermal marker (*Map2*), mesodermal marker (*Brachyury*) and endodermal marker (*Gata6*) during day 2, 4 and 6 of differentiation in Wt and KO iPSCs. The gene expression shown is relative to 18s.

**
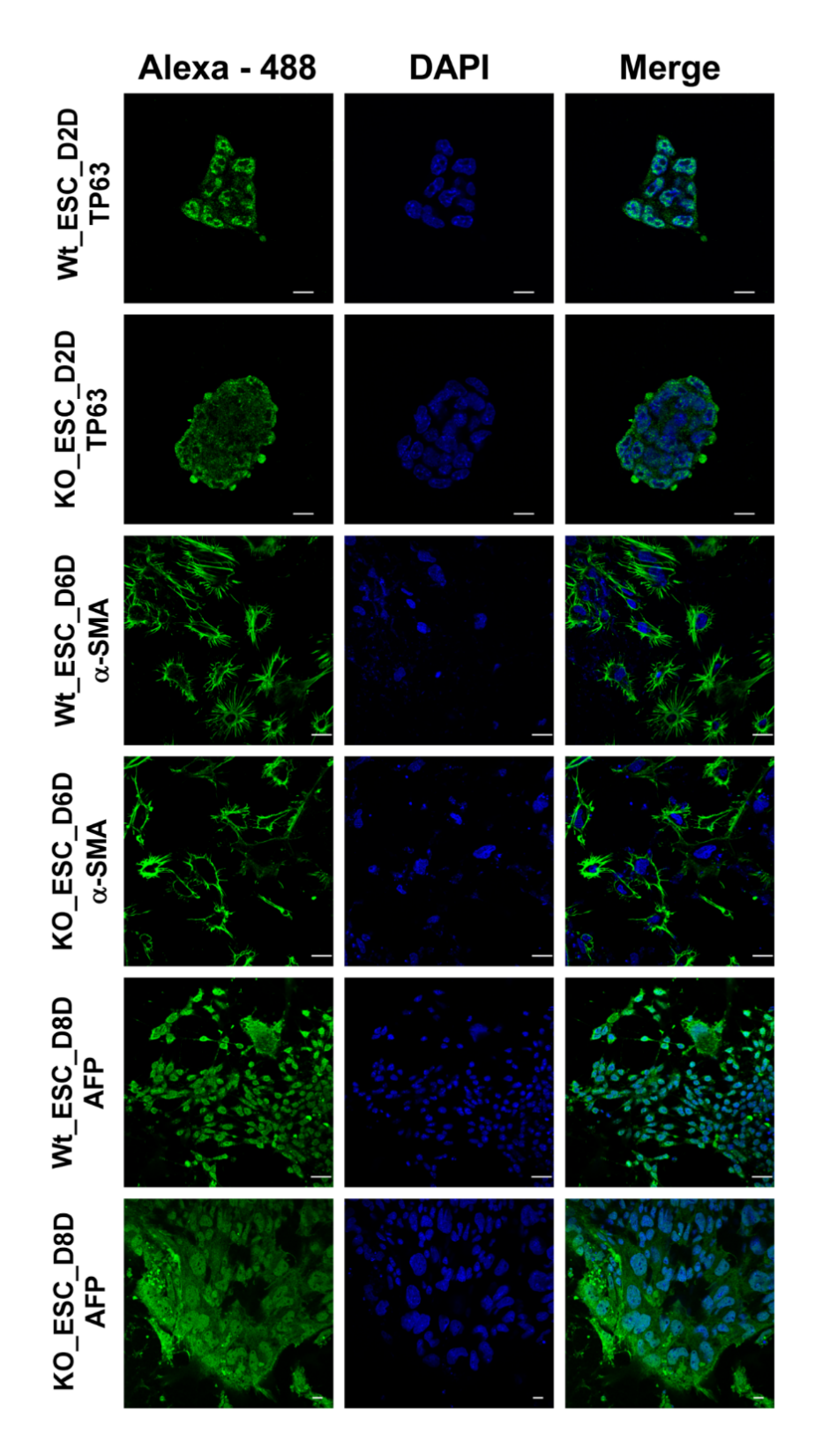
**

**Figure S11:** Immunostaining showing all three germ layer markers on day 6 of WT and KO ESC differentiation. Ectodermal marker (TP63), mesodermal marker (α-SMA) and endodermal marker (AFP).


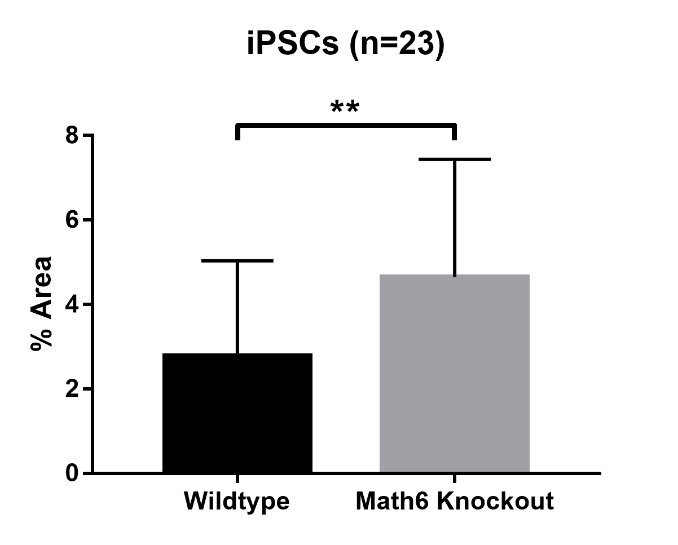


**Figure S12**: Semithin sections of WT and KO iPSCs were stained with methylene blue and studied morphometrically. The Math6 knockout iPSCs showed poor cell-cell contacts compared to WT-iPSCs. The data shown in the graph is the percentage area between cells in the iPS colonies.

## **Experimental procedures:**

**Generation of retrovirus and infection of mouse adult fibroblasts.**

Transfection was performed when the HEK 293T cells reached 50 – 70% confluence. Firstly, 10 μg of each plasmid was added (pCL Eco: pMXs vector=1:1) into 1.5 ml tube. Following, 1 ml of DMEM and 60 μl of Fugene HD reagent was added to the same tube. The DMEM/Fugene/DNA mixture was mixed thoroughly and incubated for 15 min at room temperature. Finally, the DMEM/Fugene/DNA mixture was added to the 100 mm dishes drop by drop and incubated overnight at 37 ℃ and 5% CO2. After 48 hours of transfection, the medium (viral supernatant) from each transfected dish was collected and 8 ml of fresh HEK 293T medium was added gently to each dish. The viral supernatant was filtered through a 0.45 μm syringe filter. And after 72 hours of transfection, the viral supernatant was again collected (for the second infection) and filtered using a 0.45 μm syringe filter while disposing of the HEK 293T cells. The viral supernatant that was collected after 48- and 72-hours post-transfection was subjected to ultracentrifugation at 25000 rpm for 90 min and harvested the viral pellet. The viral pellet was dissolved in 300 μl of DMEM and froze at -80^o^C until further use.

**Transduction of mouse adult fibroblasts.**

In order to reprogram MAF (mouse adult fibroblast) cells, the above-generated retrovirus was used to transduce MAF cells. One day before transduction, MAF cells (passage 1 or 2) were seeded in a 100 mm dish at a density of 8X10^5^ cells per plate in MAF Medium. Upon reaching 50% confluence on the following day, MAF cells were subjected to transduction. MAF fibroblasts (WT & KO) were infected with 150 μl of the concentrated virus per each 100 mm plate (primary infection) and further supplemented with polybrene solution at a final concentration of 8μg/ml to improve the efficiency of infection. The plates were then gently mixed and incubated for 6 hours at 37^o^C. After 6 hours, the media was changed with fresh MAF medium. On day 2, the MAF cells were again infected with the concentrated virus as the second round of infection and further incubated for 6 hours. After 6 hours, the medium was changed with fresh MAF medium and continued to incubate the cells at 37^o^C.

**Plating of Infected MAF cells on to feeder (MEF) and Matrigel (Feeder-free).**

On day 4, MAF cells were passaged on to inactivated MEF feeder or 4% Matrigel dishes in case of feeder-free cultures. The infected MAF cells were trypsinized and counted using hemacytometer and seeded onto fresh MEF feeder or 4% Matrigel-coated dishes at a density of 10,000 cells/cm^2^. The cells were then supplemented with LIF.

**Culturing iPSCs and ESCs**

iPSCs and ESCs were cultured in 2i/Lif media. Cells were passaged once every four days using TRYPLE reagent (Invitrogen). The seeding density used during passage is 1000 cells/cm^2^. Rock inhibitor was supplemented to the media for the first 12 hours after passage.

**Differentiation of iPSCs and Differentiation**

For differentiation, iPSCs and ESCs were seeded at a density of 10,000 cells/ cm^2^. Media was changed every day.

**List of primers used in the study**

| **Name** | **RT primers** | **Sequence** | **Amplicon length** |
| --- | --- | --- | --- |
| Oct4 | RT Oct4 F | 5' GCTTGGGCTAGAGAAGGATG 3' | 147 bp |
|  | RT Oct4 R | 5' GGCAGAGGAAAGGATACAGC 3' |  |
| Sox2 | RT Sox2 F | 5' CCCACCTACAGCATGTCCTA 3' | 120 bp |
|  | RT Sox2 R | 5' GTGGGAGGAAGAGGTAACCA 3' |  |
| c-MYC | RT Myc F | 5' GTGTCTGTGGAGAAGAGGCA 3' | 137 bp |
|  | RT Myc R | 5' GCGTAGTTGTGCTGGTGAGT 3' |  |
| Klf4 | RT Klf4 F | 5' GAACTCACACAGGCGAGAAA 3' | 143 bp |
|  | RT Klf4 R | 5' AAAGGCCCTGTCACACTTCT 3' |  |
| Nanog | RT Nanog F | 5' AGGGTCTGCTACTGAGATGCTCTG 3' | 364bp |
|  | RT Nanog R | 5' CAACCACTGGTTTTTCTGCCACCG 3' |  |
| E-Cadherin | RT Cdh1 F | 5' GACTGTGAAGGGACGGTCAAC 3' | 151 bp |
|  | RT Cdh1 R | 5' CCACCGTTCTCCTCCGTAGA 3' |  |
| Snail1 | RT SNAIL1 F | 5' GCCGGAAGCCCAACTATAGCGA 3' | 469 bp |
|  | RT SNAIL1 R | 5' TTCAGAGCGCCCAGGCTGAGGTACT 3' |  |
| mMath6 | RT mMath6 F | 5' CGTCAATTTCACACGTAA 3' | 430 bp |
|  | RT mMath6 R | 5' AAGAGACTCACAGTGGTGTCT 3' |  |
| Smad2 | RT Smad2 F | 5' CTTGGATTTGCAGCCAGTTA 3' | 128 bp |
|  | RT Smad2 R | 5' TGAAGCCGTCTACAGTGAGC 3' |  |
| Smad3 | RT Smad3 F | 5' GCACAGCCACCATGAATTAC 3' | 120 bp |
|  | RT Smad3 R | 5' GGAGGTAGAACTGGCGTCTC 3' |  |
| Smad4 | RT Smad4 F | 5' TGTGATCTATGCCCGTCTGT 3' | 148 bp |
|  | RT Smad4 R | 5' AATTCCAGGTGAGACAACCC 3' |  |
| Twist 1 | RT Twist1 F | 5' ACTCCAAGATGGCAAGCTG 3' | 70 bp |
|  | RT Twist1 R | 5' ATCCTCCAGACGGAGAAGG 3' |  |
| Twist2 | RT Twist2 F | 5’ CCAGAGCGACGAGATGGACA 3’ | 79 bp |
|  | RT Twist2 R | 5’ CACGGAGAAGGCGTAGCTGA 3’ |  |
| Zeb1 | RT Zeb1 F | 5‘ GCAAGCGCTTCTCACACTCC 3‘ | 149 bp |
|  | RT Zeb1 R | 5‘ AACATGCTCAGTCGCCAGGA 3‘ |  |
| Zeb2 | RT Zeb2 F | 5’CACACTCGGGCTCCTACTCG 3‘ | 148 bp |
|  | RT Zeb2 R | 5‘ TGCAGGTAAGCCCGGTTCAT 3‘ |  |
| GAPDH | RT GAPDH F | 5' CAGCCTCGTCCCGTAGACA 3' | 254bp |
|  | RT GAPDH R | 5' CGCTCCTGGAAGATGGTGAT 3' |  |
| m18S | RT m18S F | 5' GCAATTATTCCCCATGAACG 3' | 123bp |
|  | RT m18S R | 5' GGCCTCACTAAACCATCCAA 3' |  |
| N-Cadherin | RT Cdh2 F | 5’ CGTCCACCTTGAAATCTGCT 3’ | 96 bp |
|  | RT Cdh2 R | 5’ AAGGACAGCCCCTTCTCAAT 3’ |  |
| Map2 | RT Map2 F | 5' CATCGCCAGCCTCGGAACAAACAG 3' | 262 bp |
|  | RT Map2 R | 5' TGCGCAAATGGAACTGGAGGCAAC 3' |  |
| Brachyury | RT Brachyury F | 5' GCTTCAAGGAGCTAACTAACGAG 3' | 117 bp |
|  | RT Brachyury R | 5' CCAGCAAGAAAGAGTACATGGC 3' |  |
| Gata6 | RT Gata6 F | 5' ACCTTATGGCGTAGAAATGCTGAGGGTG 3' | 334 bp |
|  | RT Gata6 R | 5' CTGAATACTTGAGGTCACTGTTCTCGGG 3' |  |
| Slug/Snail2 | RT Slug F | 5' CACATTCGAACCCACACATT 3' | 130 bp |
|  | RT Slug R | 5' TATTGCAGTGAGGGCAAGAG 3' |  |
| Smuc/Snail3 | RT Smuc F | 5' TCTCGAGTCCCAGGTAGCTT 3' | 149 bp |
|  | RT Smuc R | 5' AGGCTGGCATACTCCTTGTC 3' |  |
| Stella/DPPA3 | RT Stella F | 5’ CGGGGTTTAGGGTTAGCTTT 3’ | 127 bp |
|  | RT Stella R | 5’ AAAGTCGACCCAATGAAGGA 3’ |  |
| FGF5 | RT Fgf5 F | 5’ CGCGGACGCATAGGTATTAT 3’ | 133 bp |
|  | RT Fgf5 R | 5’ ACGAGGAGTTTTCAGCAACAA 3’ |  |
| Rex1 | RT Rex1 F | 5’AAGCTGCCAGCCAGTAACC 3‘ | 220 bp |
|  | RT Rex1 R | 5’ CCTTGCGTTCCACCAACTTTC 3’ |  |
| Sox17 | RT Sox17 F | 5’ GATGCGGGATACGCCAGTG 3’ | 133 bp |
|  | RT Sox17 R | 5’ CCACCTCGCCTTTCACCTTTA 3’ |  |

**List of Antibodies used in the study** (*p=Primary antibody, s=Secondary antibody)

| **S.No** | **Antibody** | **Concentration** | **Company** |
| --- | --- | --- | --- |
| 1 | Anti-flag antibody (p) | 1:500 | Sigma – F1804 |
| 2 | Mouse anti-Oct-4 (POU5F1) conjugated with Alexa Fluor® 488 | 1:100 | Merck Millipore (MAB 441944) |
| 3 | Mouse anti-SSEA-1 conjugated with Cy3 | 1:100 | Merck Millipore (MAB 430103) |
| 4 | Mouse anti E-Cadherin (p) | 1:100 | Cell signaling (4A2 # 14472) |
| 5 | Mouse anti Oct4 (POU5F1) (p) (WB) | 1:1000 | Santa Cruz (SC 5279) |
| 6 | Anti TP63 (p) | 1:250 (IC)1:2000 (WB) | Biorbyt |
| 7 | Anti Tbx6 (p) | 1:1000 | Abcam (Ab 38883) |
| 8 | Anti Gata4 (p) | 1:250 (IC), 1:1000(WB) | Santa Cruz (SC 25310) |
| 9 | Anti Smooth muscle actin (p) | 1:200 | Sigma |
| 10 | Anti-α-Tubulin antibody(p) | 1:5000 | Sigma – T9026 |
| 11 | Anti-rabbit IgG conjugated with HRP (s) | 1:10000 | Cell signaling |
| 12 | Anti-Mouse IgG conjugated with HRP (s) | 1:10000 | Jackson immune research (315-035-008) |
| 13 | Anti rabbit IgG, Alex 488 (s) | 1:1000 | Invitrogen (A21206) |
| 14 | Anti mouse IgG, Alex 568 (s) | 1:1000 | Invitrogen (A10037) |

**List plasmids used in the study**

| **S.No** | **Name of the plasmid** | **Source** |
| --- | --- | --- |
| 1 | pCL – Eco | Addgene (12371) |
| 2 | pMXs-Oct4 | Addgene (13366) |
| 3 | pMXs-Sox2 | Addgene (13367) |
| 4 | pMXs-Klf4 | Addgene (13370) |
| 5 | pMXs-c-Myc | Addgene (13375) |
| 6 | pMXs-GFP | Generated inhouse |

**List of other material used in the study**

| **S.No** | **Item** | **Company** |
| --- | --- | --- |
| 1 | Matrigel | Corning (353234) |
| 2 | CHIR99021 | Sigma (SML1046) |
| 3 | PD03259010 | Sigma (PZ0162) |
| 4 | SB-431542 | Stem cell technology (72234) |
| 5 | TGFb1 | Peprotech (100-21) |
| 6 | LIF | Stem cell technology (78055) |

**Karyotypes of the iPS cells**

**
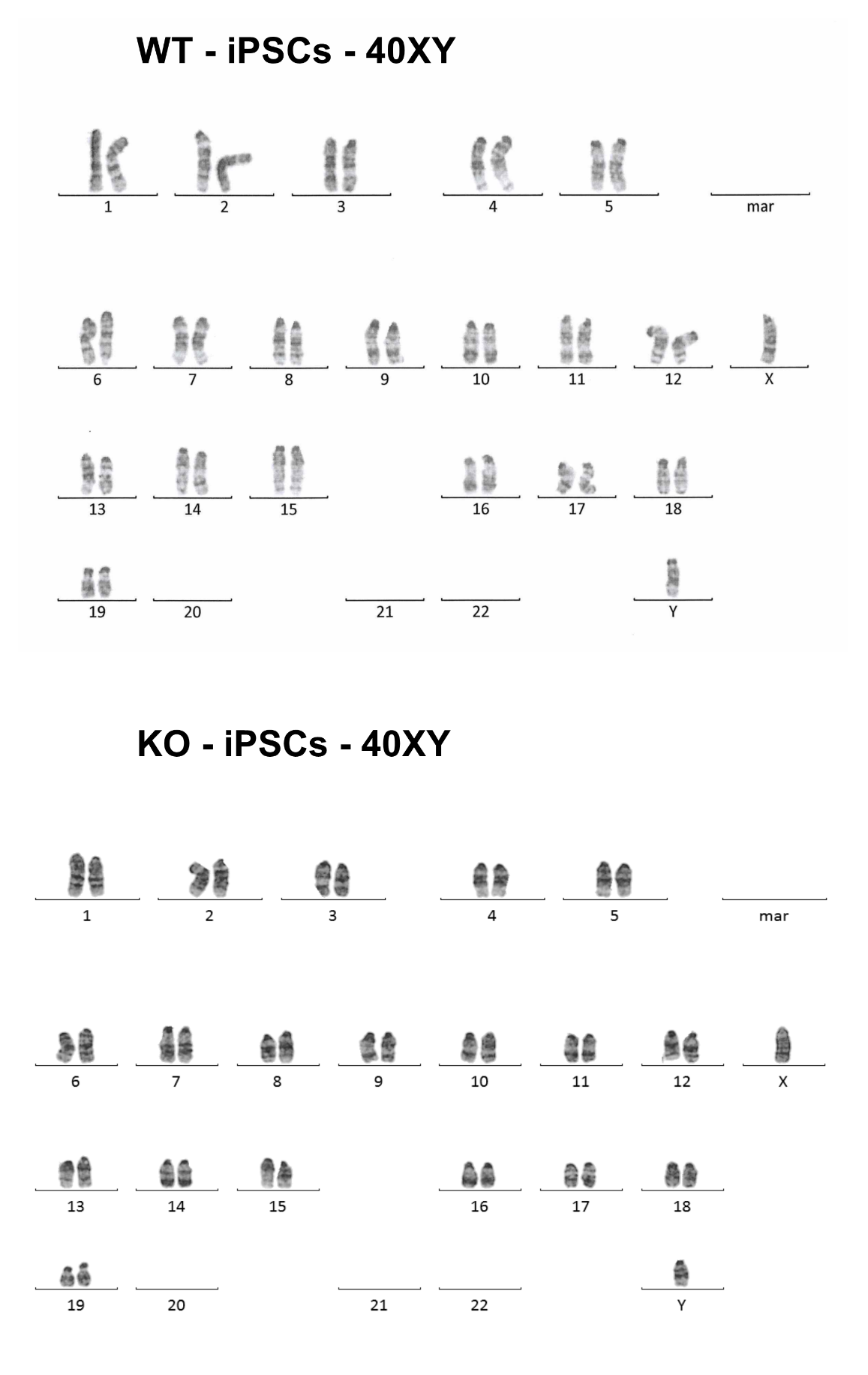
**

**Karyotypes of the ES cells**

**
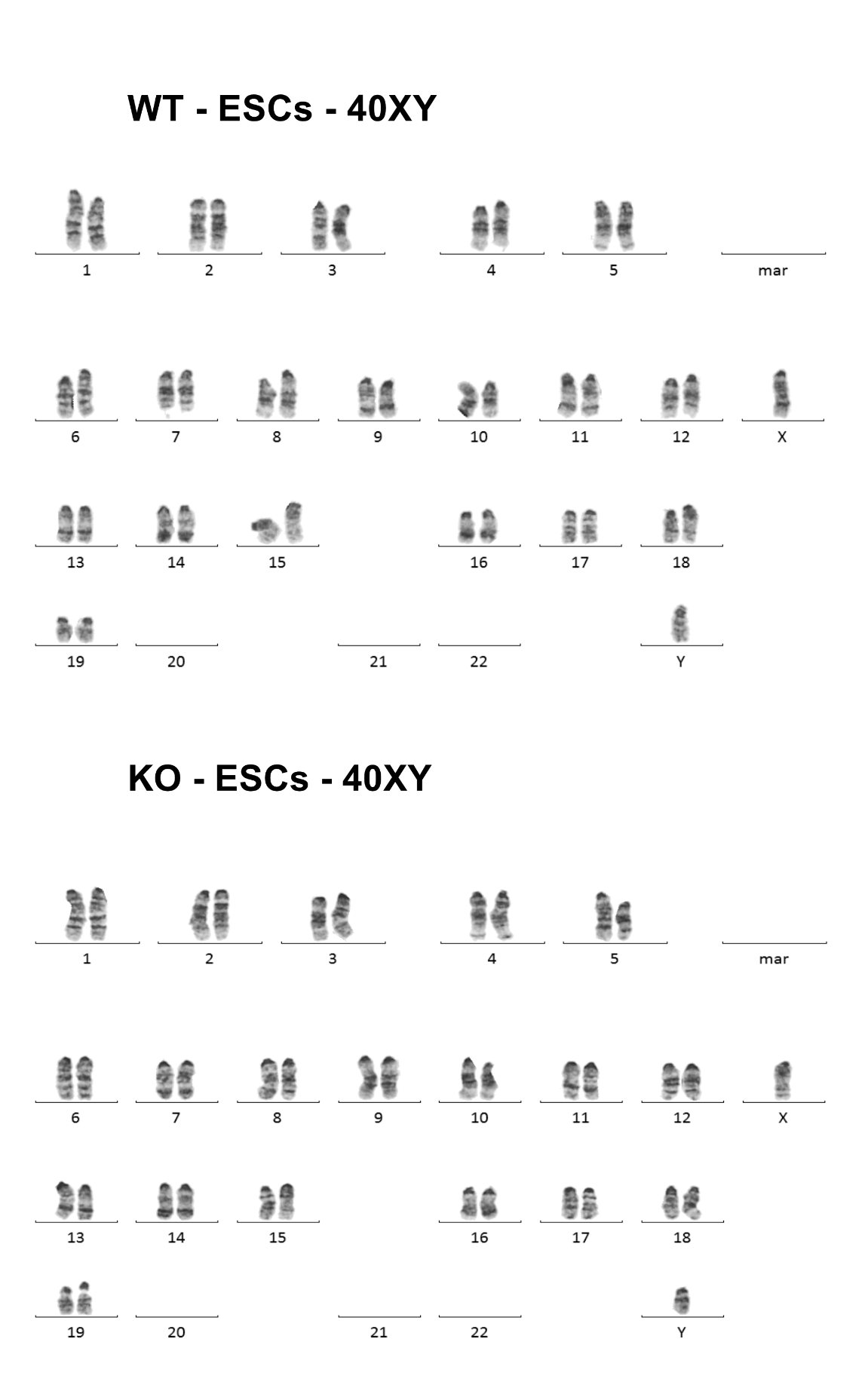
**
